# Assessing the views and opinions of psychiatric patients receiving genome-guided treatment within the scope of the PREPARE preemptive pharmacogenomics clinical study

**DOI:** 10.3389/fphar.2025.1722339

**Published:** 2025-11-21

**Authors:** Margarita-Ioanna Koufaki, Maria-Theodora Pandi, Maria Skokou, Kariofyllis Karamperis, Konstantinos Vasileiou, Christina Mitropoulou, Marc S. Williams, George P. Patrinos

**Affiliations:** 1 Department of Pharmacy, School of Health Sciences, University of Patras, Patras, Greece; 2 The Golden Helix Foundation, London, United Kingdom; 3 Department of Genetics and Genomics, College of Medicine and Health Sciences, United Arab Emirates University, Al-Ain, United Arab Emirates; 4 Geisinger, Danville, PA, United States; 5 Hellenic Pasteur Institute, Athens, Greece; 6 Zayed Center for Health Sciences, United Arab Emirates University, Al-Ain, United Arab Emirates; 7 Clinical Bioinformatics Unit, Department of Pathology, Faculty of Medicine and Health Sciences, Erasmus University Medical Center, Rotterdam, Netherlands

**Keywords:** pharmacogenomics, psychiatric patients, questionnaire, attitudes, intentions to adopt, PREPARE

## Abstract

**Introduction:**

Pharmacogenomics (PGx), an essential component of Personalized Medicine (PM), holds the potential to transform modern medical practice. While significant efforts have been made to enhance genomics education among healthcare professionals and explore the perspectives of various stakeholders, the understanding of patients’ views on PGx remains relatively limited.

**Methods:**

Here we present findings from a cohort of 201 patients with various psychiatric illnesses enrolled in the PREPARE prospective PGx study, aiming to investigate their opinions and perceptions regarding the clinical adoption of PGx.

**Results:**

Although, 84% of respondents demonstrated a low level of PGx awareness and available tests, 99.5% strongly agreed that PGx testing reduces the occurrence and severity of adverse drug reactions (ADRs) and improves decision-making for prescribing the most appropriate medication. Furthermore, 42.45% of patients who had previously experienced an ADR were significantly more likely to accept a tailored treatment plan based on physician guidance (p < 0.001), compared to 27.37% of those without ADRs.

**Discussion:**

Collectively, our data highlights the need for enhanced patient education and awareness about PGx testing and reflects an overall positive patient attitude towards genome-guided therapeutics.

## Introduction

Personalized Medicine (PM) has gained considerable attention as an innovative healthcare strategy. This pioneering approach adapts the therapeutic regimen of everyone based on their personal genomic variants, supporting more precise and predictive prescribing and reducing the reliance on empiric approaches based on population data. Significant efforts have been made in recent years to increase awareness and educate stakeholders on the various aspects of PM, with particular emphasis on pharmacogenomics (PGx), due to its broad clinical applications.

More precisely, several initiatives have been implemented worldwide, encompassing information campaigns, seminars, and training programs. The most noticeable example is the integration of PGx into the curricula of several universities across the world, incorporating sessions, lectures, and comprehensive courses focused on PGx. This tendency aims to enrich genomics education and training of the future generations of healthcare professionals, i.e., physicians, pharmacists, etc., by equipping them with the necessary knowledge to effectively apply PM in their respective fields (Patrinos and Katsila, 2016), as this would potentially lead to an increase in the adoption rate of PGx testing in the clinical practice (Koufaki et al., 2023). Additionally, use of point-of-care “just in time” interventions within the electronic health record and pharmacist-directed PGx care has augmented traditional educational approaches with some success (Caraballo et al., 2020).

Although significant attention has been given to clinical stakeholders, the level of information aimed at patients, caregivers, and relatives has been understudied. Most of the published research is mainly focused on physicians’ or pharmacists’ perceptions, attitudes, and opinions, with very limited focus on public or patients’ awareness and understanding (Allen et al., 2022). Patients are key stakeholders in the PGx process flowchart that should not be neglected, as they are the end-users of this technology, but it seems that the great majority of the general public is not aware of PGx testing and its role in drug treatment (Daud et al., 2017; Gawronski et al., 2023; Naik et al., 2009). Indeed, according to Cuffe and coworkers (2014), 20% of cancer patients did not fully understand the purpose of genetic and PGx testing and were concerned about their application (Cuffe et al., 2014), while in Trinidad and coworkers (2015), participants claimed that they had observed differences in drug response among individuals, but they were not familiar with the idea of genetic association related to it (Trinidad et al., 2015).

Nonetheless, patients’ awareness and attitude can be changed if they receive proper information and exhibit a good grasp of PGx concepts and their potential benefits (Daud et al., 2017; Gawronski et al., 2023; Haga et al., 2016). Evidently, patients with depression who had received clear information by reviewing an educational video had a better level of knowledge and awareness compared to a group without exposure to information (Sloat et al., 2022), while in the Gawronski and coworkers (2023) study, researchers demonstrated that 61% of patients expressed their interest in undergoing PGx testing upon being properly informed (Gawronski et al., 2023).

Several studies have been performed in the past attempting to shed light on the general public’s genomic literacy and perception of PGx testing, but they are mainly focused on oncology patients and in non-European clinical settings. Here, we aimed to investigate the informedness and behaviour, general preferences, opinions, and attitudes of psychiatric patients in Greece, who participated in the PREemptive Pharmacogenomic testing for preventing Adverse drug REactions (PREPARE) study, a multinational, multisite, prospective PGx clinical study aiming to compare PGx-guided treatment towards conventional therapy.

## Materials and methods

### Survey design and development

To investigate the perceptions of psychiatric patients for PGx testing, we employed a rigorous, multistage survey development process. In the beginning, an extensive literature review was conducted to indicate the factors that were highlighted in previous studies in PM, PGx, and clinical genetic testing. The items used were inspired and adapted to this study’s needs from previous studies in the patients’ population. To establish content validity, the draft survey was reviewed by one professor of PGx and two physicians, who had direct experience with PGx implementation. A survey instrument was developed to assess patients’ perceptions, knowledge, and attitudes towards PGx testing. This questionnaire was specifically designed to be employed in a clinical setting and to be administered before PGx testing. We conducted a pilot test with 15 patients and lay people to evaluate item clarity, relevance, and overall survey coherence.

The descriptive cross-sectional survey was conducted from May 2017 to October 2018. This study used a 28-item questionnaire developed by the Laboratory of Pharmacogenomics and Individualized Therapy at the Department of Pharmacy, University of Patras, Greece, as part of the PREPARE study (ClinicalTrials.gov NCT03093818). PREPARE was launched in March 2017, as part of the European Commission-funded Ubiquitous Pharmacogenomics (U-PGx; www.upgx.eu) project (Van Der Wouden et al., 2017; Skokou et al., 2024). The PREPARE study was one of the first investigator-initiated, open-label, multicenter, cluster-randomized crossover implementation studies that focused on investigating the effectiveness of preemptive PGx testing in preventing adverse event reactions. The study’s protocol has been published previously (Skokou et al., 2024; Swen et al., 2023).

The questionnaire (see complete questionnaire in the Supplementary Material 1) consisted of seven main sections, including general level of knowledge related to PGx interventions (3 questions), perceived PGx benefit (6 questions), general preferences (2 questions), informedness and behavior (7 questions), barriers and concerns towards to PGx (3 questions), willingness to adopt PGx in clinical practice (5 questions), overall satisfaction from PGx testing (2 questions). Most of the items were measured on a 4-point scale, with 1 corresponding to “totally disagree” and 4 corresponding to “totally agree”. Other scales used include a 5-point scale (1- Not at all; 5 – Absolutely), used for two items related to informedness, behaviour, and intentions and four items related to willingness to adopt, a scale from 0 (Not at all) to 10 (Absolutely), that was used for all the items related to overall satisfaction, as well as 3-point scale (e.g., Yes; No; I do not remember).

The study was approved by the Institutional Review Board of Patras University General Hospital (825/28.12.2016). Informed consent was provided to all participants, to be signed before proceeding with the completion of the questionnaire. Physicians have previously explained to all patients what PGx is, its application, potential benefits, and risks, along with the process of PGx testing in great detail in the context of their participation in the PREPARE study. All participants’ questions were answered and clarified prior to completing the survey.

### Study sample

The study population consisted of 646 outpatient psychiatric patients participating in the PREPARE study in a Greek site who were recruited as part of the genome-guided arm and were about to undergo PGx testing. Participants were over 18 years old, had a diagnosis of a psychiatric disorder based on the International Classification of Diseases, 10th Revision (ICD-10), ranging from schizophrenia to bipolar disorder, and were under psychiatric medication treatment. The questionnaire was distributed in paper form to all patients who consented to participate in the PREPARE study. Only 201 of them gave their written consent to complete it.

The survey was completed in two phases. In the first phase, participants were asked to fill out the survey’s questions related to demographic information, level of knowledge, perceived PGx benefit and general preferences before getting a PGx test, while in the second phase, participants were asked to answer questions related to informedness and behaviour, barriers and concerns, overall satisfaction and willingness to adopt upon determining their treatment scheme based on PGx results.

### Data analysis

All statistical analyses were carried out using R version 4.5.1 (Swen et al., 2023). At first, the questionnaire was assessed for internal consistency, as a whole and within each section, by computing Cronbach’s alpha with the psych R package, while allowing the scales of items that were negatively correlated with the first principal component to be automatically reversed (Revelle, 2025). In addition, the baseline characteristics of those who participated in this survey were evaluated in terms of their representativeness towards the whole cohort of the PGx arm.

Moreover, continuous variables, i.e., baseline patient characteristics, are reported as median values with interquartile ranges (IQRs), while categorical variables are represented using absolute and relative frequencies. Survey responses were analyzed descriptively and visualized using bar plots generated for every group of related items using the ggplot2 package (Wickham, 2016). To examine correlations between questionnaire items, the non-parametric Spearman correlation coefficient (rs or rho (ρ)) between the different questions was computed using the psych package in R (Barber and Thompson, 2000). Responses were numerically encoded, and items with zero variance across participants were excluded both from correlation analysis and any inference statistics [more specifically, this refers to the following 2 questions: “I worried that PGx testing will impact me financially. (1 = Disagree, 4 = Agree)” and “I have all necessary information to understand how PGx works as a pharmacotherapy tool. (1 = Disagree, 4 = Agree)”].

Pairwise correlation strength was classified according to the absolute value of the coefficient (|ρ|) as “strong” (|ρ| = 0.60–0.79) or “very strong” (|ρ| ≥ 0.80), following conventions used in social science research. This analysis was exploratory in nature, and no adjustment was made for potential confounding factors (e.g., age, diagnosis, prior adverse drug reactions (ADRs)); such influences are therefore acknowledged as a limitation. Pairwise correlation coefficients, along with their respective statistical significance, before and after Benjamini–Hochberg (BH) correction for multiple comparisons, as provided by the corr. test function from the psych package (see Supplementary Material 2).

The impact of selected demographic factors (age, gender, level of education, prior experience with ADRs, and patient’s diagnosis) on questionnaire responses was assessed using hypothesis testing. Age was encoded as a categorical variable with the following levels: 18–34, 35–44, 45–54, 55–64, and >65 years. Depending on the number and distribution of the available responses, associations between questionnaire responses and demographics were tested using Pearson’s chi-square test or Fisher’s exact test. Descriptive summaries and group comparisons were generated with the gtsummary package in R (Sjoberg et al., 2021). All tests were two-sided, and the significance level (α) was set to 0.05. To account for multiple comparisons, p-values were adjusted using Benjamini–Hochberg correction to control the false discovery rate, as implemented in gtsummary via R’s statsp.adjust() function.

## Results

Our sample population exhibited balance in terms of gender and age distribution, as shown in Table 1. In particular, the average participants’ age was 48 years old, and the sample consisted of 102 males and 99 female participants. 31% of the respondents have graduated from high school, 26% had completed only primary school education, and 25% were B.Sc. degree holders. Participants’ quality of life was characterized as fair by 43% followed by a poor level of life in approximately 25% of cases. This sample is representative of the wider cohort of participants in the PGx arm in the PREPARE trial. Upon comparing the baseline characteristics of the questionnaire subgroup (n = 201) against the entire PGx arm (n = 646) across demographic, clinical, lifestyle, and pharmacogenetic variables, we identified no statistically significant differences between the two groups (Supplementary Tables S1-S2).

**TABLE 1:** Patient demographics.

	Overall N = 201^a^	Female N = 99^a^	Male N = 102^a^
Age	48 (38, 58)	49 (40, 58)	47 (37, 57)
Indication Category			
Anxiety disorders	7 (3.5%)	4 (4.0%)	3 (2.9%)
Bipolar disorder	40 (20%)	22 (22%)	18 (18%)
Major depression	89 (44%)	51 (52%)	38 (37%)
Other	15 (7.5%)	6 (6.1%)	9 (8.8%)
Schizophrenia	50 (25%)	16 (16%)	34 (33%)
Education level			
Primary school	52 (26%)	29 (29%)	23 (23%)
Junior high school	35 (17%)	18 (18%)	17 (17%)
High school	63 (31%)	27 (27%)	36 (35%)
Bachelor	50 (25%)	24 (24%)	26 (25%)
MSc	1 (0.5%)	1 (1.0%)	0 (0%)
Level of life			
Poor	57 (28%)	29 (29%)	28 (27%)
Fair	88 (44%)	38 (38%)	50 (49%)
Good	54 (27%)	31 (31%)	23 (23%)
Very good	1 (0.5%)	0 (0%)	1 (1.0%)
Excellent	1 (0.5%)	1 (1.0%)	0 (0%)
Family status			
Single	88 (44%)	30 (30%)	58 (57%)
Married	64 (32%)	31 (31%)	33 (32%)
Divorced	34 (17%)	24 (24%)	10 (9.8%)
Widow/Widower	15 (7.5%)	14 (14%)	1 (1.0%)
Smoking status			
Current	114 (57%)	50 (51%)	64 (63%)
Ex-smoker	16 (8.0%)	4 (4.0%)	12 (12%)
Non-smoker	71 (35%)	45 (45%)	26 (25%)
Alcohol consumption (units/week)			
<1	146 (73%)	84 (85%)	62 (61%)
1–5	37 (18%)	11 (11%)	26 (25%)
6–14	9 (4.5%)	2 (2.0%)	7 (6.9%)
15–21	6 (3.0%)	2 (2.0%)	4 (3.9%)
22–49	1 (0.5%)	0 (0%)	1 (1.0%)
>50	2 (1.0%)	0 (0%)	2 (2.0%)
Level of health			
Weak	93 (46%)	50 (51%)	43 (42%)
Satisfactory	51 (25%)	27 (27%)	24 (24%)
Good	43 (21%)	16 (16%)	27 (26%)
Very good	12 (6.0%)	5 (5.0%)	7 (7.0%)
Excellent	2 (1.0%)	1 (1.0%)	1 (1.0%)
Number of concomitant medications	3 (2, 5)	4 (2, 6)	3 (2, 5)

^a^ Median (IQR); n (%).

### Internal consistency

Internal consistency of the questionnaire was examined using Cronbach’s alpha. The overall questionnaire demonstrated acceptable reliability (α = 0.75), however, the reliability estimates for the individual subscales varied considerably (Supplementary Table S3). More specifically, Barriers and Concerns showed excellent internal consistency (α = 0.96), while both Level of Knowledge (α = 0.85) and Overall Satisfaction (α = 0.87) demonstrated good reliability. In addition, the Willingness to Adopt subscale showed acceptable reliability (α = 0.70). In contrast, Perceived Benefits (α = 0.55), Informedness and behaviour (α = 0.26), and General Preferences (α = 0.039) demonstrated poor internal consistency, suggesting that the items within these domains may not consistently measure the same underlying construct. Moreover, eight pairs of questions showed strong or very strong positive correlations after BH correction. The strongest were between concerns about unauthorized use of PGx information and data confidentiality (ρ = 0.93), and between willingness to recommend PGx testing to children and to friends/relatives (ρ = 0.91). Familiarity with PGx also correlated with awareness of its existence (ρ = 0.81). Willingness to recommend PGx testing (to either one’s children: ρ = 0.69, or to friends/relatives: ρ = 0.64) was associated with the likelihood of following physician-recommended PGx-guided treatments. In addition, satisfaction with PGx testing was linked to perceived utility in healthcare decisions (ρ = 0.75), preference for testing before prescriptions to belief in reduced ADRs (ρ = 0.70) and stopping medication for ineffectiveness to noticing ADRs (ρ = 0.67). Together, these findings underscore the interrelatedness of patient attitudes toward PGx testing and its perceived value.

### Responders’ attitudes towards PGx

**FIGURE 1:**
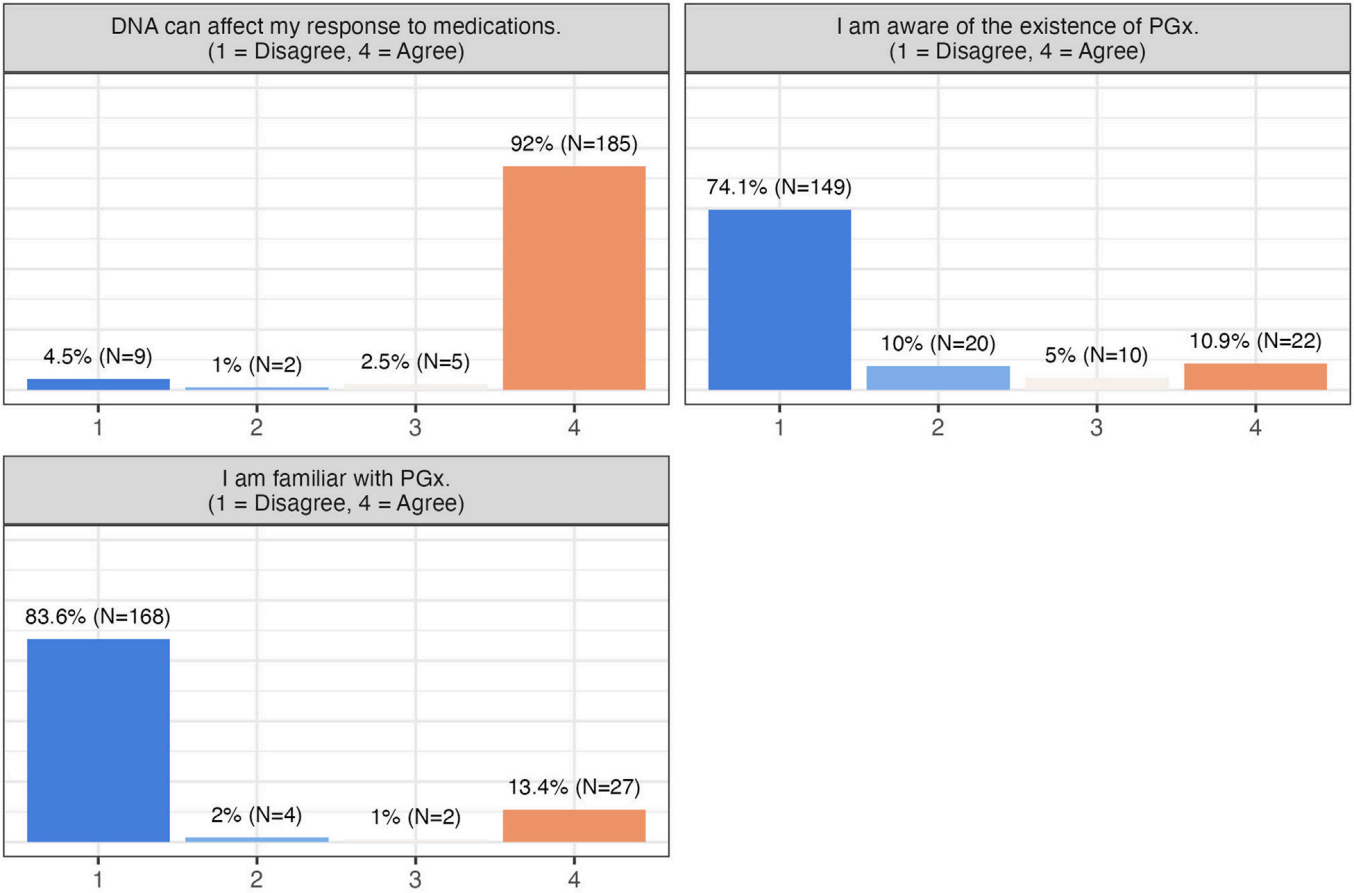
Participants’ level of awareness.

Participants in this study expressed strong satisfaction and positive attitudes toward PGx testing, with over 90% rating their experience with a score of 8 or higher on a scale of 0 (not satisfied with my PGx experience at all) to 10 (absolutely satisfied). PGx testing was perceived as highly useful in guiding pharmacotherapy decisions, and most participants preferred undergoing testing prior to starting medication. They were also receptive to further education on PGx (Table 2), despite relatively low baseline awareness (84% reported being unfamiliar with PGx), while most correctly recognized that DNA can affect drug response (94.5%) (Figure 1).

**TABLE 2:** Attitudes, opinions and perceptions towards PGx testing. Questions related to general preferences were asked before the pharmacogenetic testing, while overall satisfaction related questions were asked after the pharmacogenetic testing was applied.

	Overall, N = 201^a^	Female, N = 99^a^	Male, N = 102^a^
General preferences			
Would you prefer to have PGx testing before being prescribed the index drug?			
Moderately agree	2 (1.0%)	2 (2.0%)	0 (0%)
Agree	199 (99%)	97 (98%)	102 (100%)
Whose responsibility is to inform you and discuss your questions with based on your opinion?			
Physician	138 (69%)	71 (72%)	67 (66%)
Researcher/Geneticist	59 (29%)	28 (28%)	31 (30%)
Other	4 (2.0%)	0 (0%)	4 (3.9%)
Overall satisfaction			
I Am generally satisfied with my experience using PGx testing. (0 = not at all, 10 = absolutely)			
6	3 (1.5%)	1 (1.0%)	2 (2.0%)
7	7 (3.5%)	1 (1.0%)	6 (5.9%)
8	34 (17%)	17 (17%)	17 (17%)
9	60 (30%)	27 (27%)	33 (32%)
10	97 (48%)	53 (54%)	44 (43%)
PGx testing was helpful in making decisions about my health. (0 = not at all, 10 = absolutely)			
5	1 (0.5%)	0 (0%)	1 (1.0%)
7	16 (8.0%)	4 (4.0%)	12 (12%)
8	20 (10.0%)	9 (9.1%)	11 (11%)
9	52 (26%)	26 (26%)	26 (25%)
10	112 (56%)	60 (61%)	52 (51%)

^a^ n (%).

**FIGURE 2:**
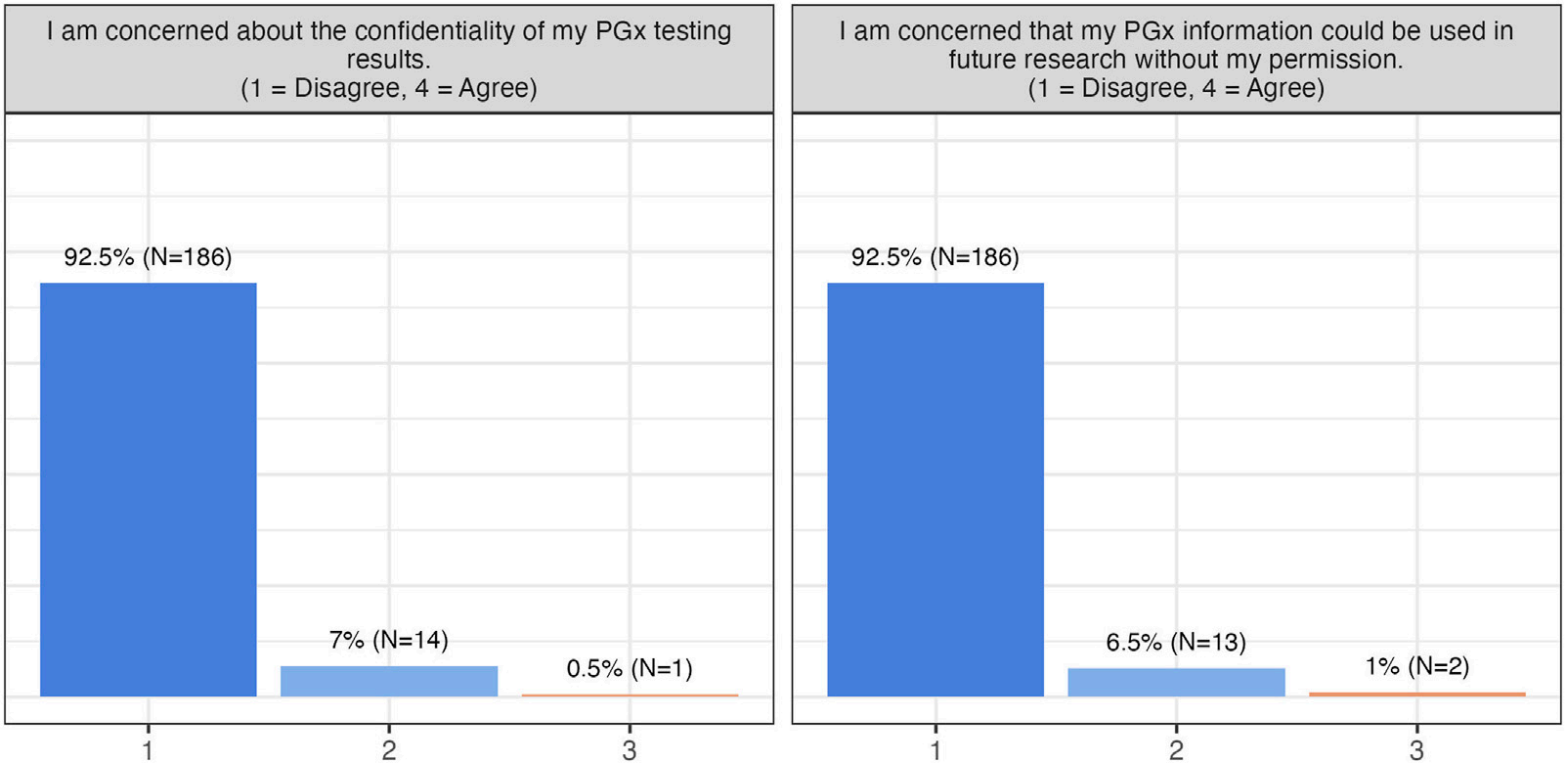
Participants’ responses towards barriers and concerns. This group of questions includes one more question (I am concerned that PGx testing will impact me financially.), to which all patients has responded “Disagree” and is thus not visualised here.

**FIGURE 3:**
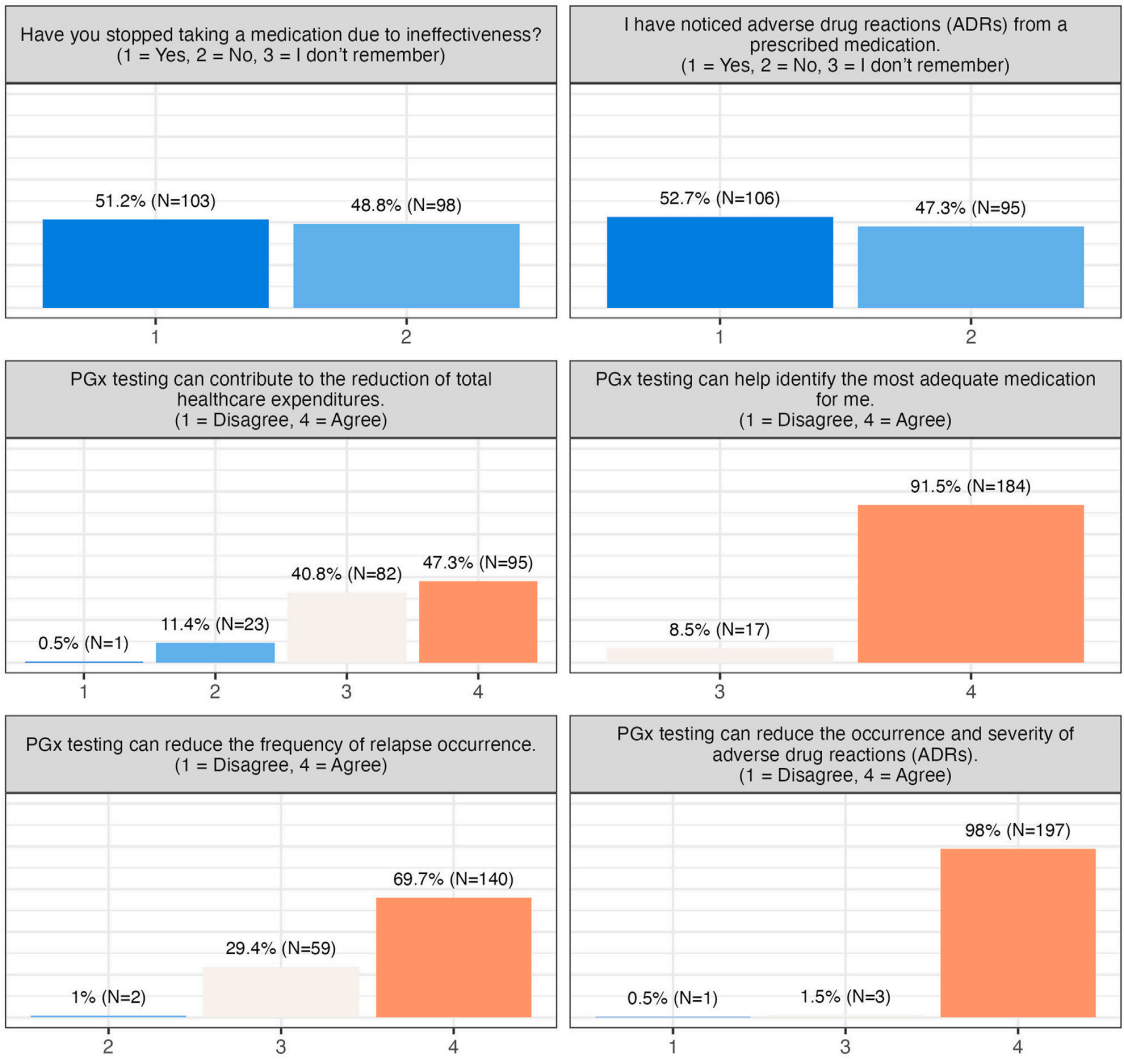
Participants’ perceived benefits of PGx testing.

About half of the participants reported experiencing ADRs, and slightly over 50% had discontinued medication due to ineffectiveness. Nonetheless, concerns about cost, reimbursement, or data privacy were minimal, and the majority were unconcerned about unauthorized use of their PGx data (Figure 2). Participants broadly recognized the clinical benefits of PGx testing, including reduced ADRs (99.5% patient agreement), improved prescribing decisions (100% patient agreement), decreased frequency of relapse (99% patient agreement), and potential healthcare cost reduction (88% patient agreement) (Figures 2, 3).

**FIGURE 4:**
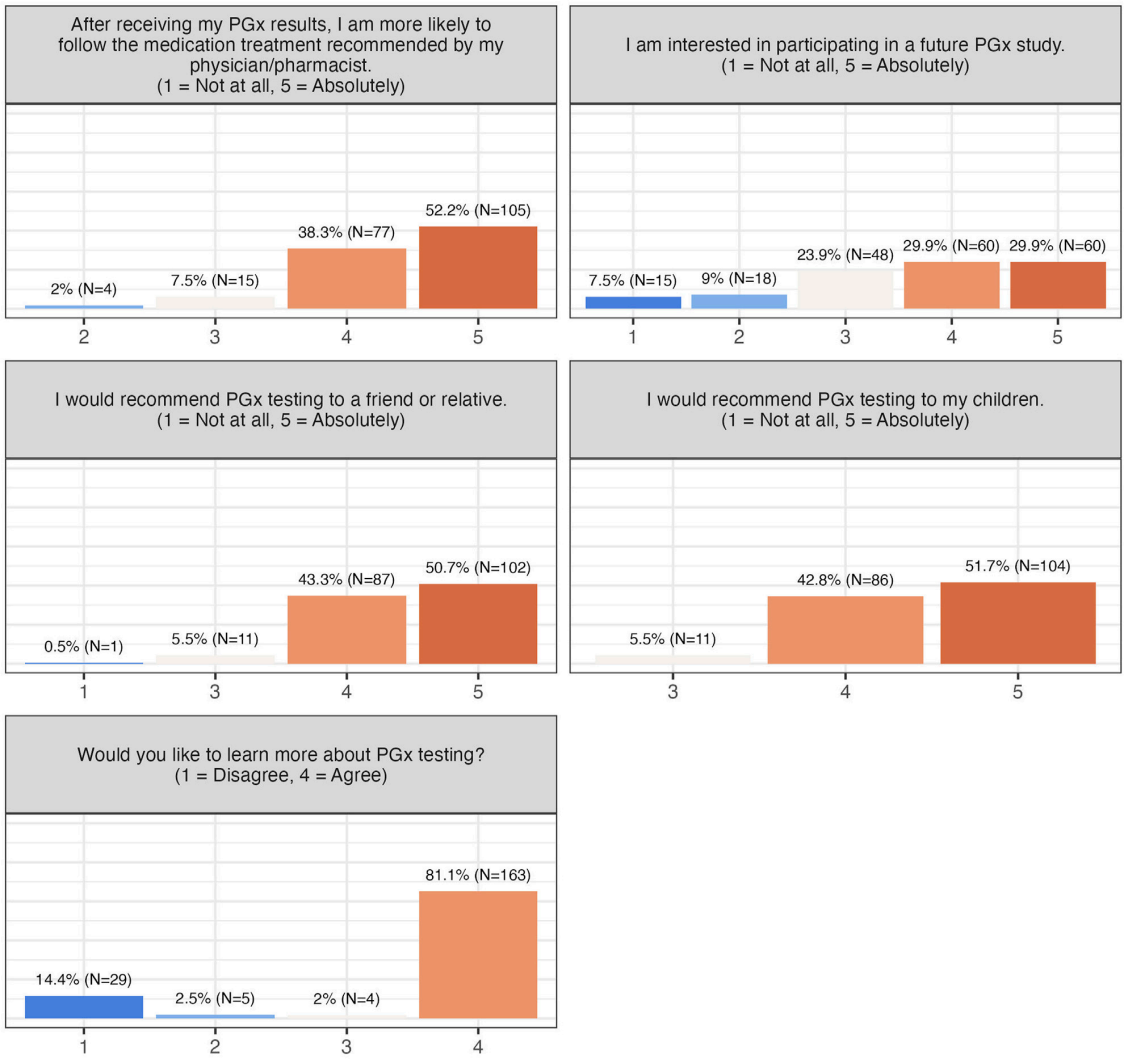
Participants’ willingness to adopt PGx testing in the future.

Willingness to adopt PGx testing and advocate for it was high: 90.5% were likely to follow physician-recommended treatments, 94%–94.5% would recommend testing to children, friends, or relatives, and 60% indicated willingness to participate in future PGx studies (Figure 4). Collectively, these findings demonstrate strong acceptance, perceived utility, and advocacy for PGx testing among participants.

Respondents’ attitudes did not drastically change upon undertaking a PGx test. Most patients (over 70%) trusted their physician to adjust medications based on PGx results, with 38% having already done so under medical guidance, while none of them were prone to change their pharmacotherapy without consulting their physician first. Overall, the respondents overwhelmingly agreed that the impact and benefits of PGx testing, as well as the information provided to them before they participated in the study were clear and comprehensive (informedness and behaviour figure, Supplementary Figure S1).

### Demographics affecting attitudes

Finally, the impact of different demographic variables (age, gender, education, psychiatric indication, prior ADRs) was assessed (see Supplementary Tables S4–S8). After BH correction, only prior experience with ADRs showed a significant effect. Patients with a history of ADRs were more likely to accept physician-guided tailored treatments (42.4% vs. 27.4%, p < 0.001, p-adj = 0.003) and to discontinue ineffective medications (83% vs. 15.8%, p < 0.001, p-adj<0.001). These findings indicate that previous ADRs increase receptiveness to therapy adjustments and discontinuation of ineffective treatments (Supplementary Table S4).

## Discussion

Implementing PGx testing in psychiatry and other medical fields is clinically advantageous for drug and patient management (Swen et al., 2023). According to Skokou and coworkers (2024), patients whose drug treatment was guided by PGx results experienced fewer ADRs and reduced hospitalization rates (Skokou et al., 2024; Pandi et al., 2025). Identifying and considering patients’ perspectives is vital in the adoption and success of PGx testing, ultimately leading to a more tailored and effective healthcare approach.

This study adds to the growing body of evidence supporting patient satisfaction and the perceived importance of PGx testing in patients with psychiatric disorders. The strength of this study lies on the following features: (a) it includes a large number of patients from different ICD-10-based psychiatric diagnoses and different medications; (b) all of the patients have been enrolled in a real-world prospective PGx study and have undertaken a PGx test, (c) all respondents have been informed by a specialist on the topic and who distributed paper questionnaires to complete. Also, this is perhaps the only study that focuses on such diverse and numerous cohorts of psychiatric patients’ knowledge, attitudes, and awareness regarding PGx as part of a real-world prospective PGx testing study (PREPARE).

Based on our results, participants’ overall experience was positive and insightful, although their level of PGx awareness was low. Participants exhibited a favorable attitude towards the application of PGx in drug management, largely due to the proper information provided by their treating physicians. Moreover, most participants recognized the significant clinical benefits of PGx testing in managing ADRs and drug efficacy, acknowledging its importance and utility in the decision-making process for personalising treatment therapy. Notably, nearly 95% of participants would recommend PGx testing to their family members or friends, indicating a high level of self-confidence and trust towards PGx intervention. This was also commented by Lemke and coworkers (2018) (Lemke et al., 2018).

The study’s findings are consistent with the literature. In particular, in another study focusing on psychiatric patients, most of the participants expressed a strong interest in receiving PGx-guided treatment, but their familiarity with the intervention was very low (Kastrinos et al., 2021). Furthermore, Lemke and coworkers (2018) study demonstrated that, almost 60% of respondents agreed that PGx testing could be a helpful tool for health-related decisions for now and in the future (Lemke et al., 2018), while in Trinidad and coworkers, 2015, interviewees in the antidepressant and carbamazepine groups pinpointed the frustration they felt during the trial and error period in identifying the right medication for them (Trinidad et al., 2015). They indicated how important it was to avoid such unpleasant challenges and get a personalized treatment in a fast and easy way (Trinidad et al., 2015). Haga and coworkers (2016) also indicated that interviewed patients expressed increased confidence in terms of the safety and efficacy of their prescribed medication when pharmacotherapy was determined based on PGx results (Haga et al., 2016). This highlights the perceived value of PGx testing among patients, as it provides a more personalized and targeted approach to their treatment, leading to greater trust in their medication’s ability to improve their condition.

Most participants were willing to adhere to the proposed therapy following PGx testing. Notably, patients tended to trust their treating physicians’ recommendations rather than those of any other healthcare professionals, a trend commonly described in the literature (Lemke et al., 2018; Pereira et al., 2019). For instance, a multinational quantitative study revealed that 75% of respondents felt more comfortable if a physician recommended and analyzed a PGx testing compared to pharmacists (Pereira et al., 2019), which was also highlighted in the Lemke and coworkers (2018) research on a US cohort (Lemke et al., 2018). Furthermore, respondents exhibited a very positive attitude and perception about PGx testing and its role in personalizing pharmacotherapy. Yet, only 16% of them were aware of the PGx field and its available interventions. This finding aligns with previous studies, where many patients who underwent PGx testing favored the intervention, despite not having been previously informed about PGx interventions (Swen et al., 2023; R Development Core Team, 2025) or being unable to accurately describe it (Allen et al., 2022). These results collectively underscore the need for improved patient education and awareness regarding PGx testing.

The studies conclude with varying results on that topic (Lemke et al., 2018; Truong et al., 2020; Doyle et al., 2023). Only 13% of the respondents in the Truong and coworkers (2020) study could recognize the term PGx but were able to answer correctly knowledge-based questions about PGx (Truong et al., 2020). Similarly, participants in Lee and coworkers (2022) study had relatively low mean scores (Lee et al., 2022). In this study, most of the participants exhibited a great deal of knowledge, since they answered correctly the relevant questions, a result that is supported by Liko and coworkers (2020) semi-structured qualitative study among psychiatric patients (Liko et al., 2020). This underscores the importance of proper information and counseling of patients with the healthcare professionals before PGx testing, a conclusion that was also mentioned in Haga and coworkers (2012) study (Haga et al., 2012).

Regarding concerns and barriers, in contrast to several other studies, almost none of the respondents raised any concern about PGx testing costs, data privacy, or confidentiality. In other research projects, participants have been worried about various aspects of PGx testing settings, including financial and ethical concerns (Melendez et al., 2023). Indeed, nearly 65% of Japanese patients have expressed significant concerns about data protection (Kobayashi et al., 2011), and two-thirds of participants in the Naik and coworkers (2023) study were uncertain about data confidentiality and feared that their data might be accessible to insurance companies or other entities (Naik et al., 2009). Moreover, the high cost of PGx testing and lack of reimbursement are commonly mentioned as potential barriers by patients and healthcare professionals. In our study, participants undertook the PGx test free-of-charge as part of an observational study in a public hospital upon signing written informed consent. Consequently, it is believed that patients felt safe and had no reason to worry about the relevant costs or data privacy. This could also be a limitation owing to the fact that it does not fully reflect the real-world setting.

Half of the respondents had experienced ADRs due to prescribed medication in the past and needed to stop it. This previous unpleasant experience was imprinted in their current attitude, a fact that was illustrated when this demographic variable was assessed. This observation justifies their willingness to undergo PGx testing, recommended to family and friends, and to show trust in the process. In a Japanese study, 55% of patients who experienced a severe ADR were more willing to donate blood samples for PGx testing compared to those not suffering one, while there was an association between willingness to undergo PGx testing and past experience of an ADR (Kobayashi et al., 2011). In the Haga and coworkers (2016) study, nine out of ten participants had stopped a medication due to an ADR, and those were more convinced about the usefulness of PGx testing in drug management (Haga et al., 2016). This finding underlies the significant impact of personal experience on an individual’s perception and self-confidence and highlights the importance of this factor.

Finally, participants expressed an interest in acquiring more knowledge about PGx technology and its clinical applications. This finding underscores the need to enhance information dissemination and increase public awareness, particularly in light of the general population’s positive attitude towards this technology. Developing targeted communication strategies for the lay public has been a topic of recent discussion (Olson et al., 2017). Based on Lee and coworkers (2022), 80% of the respondents showed an eagerness to gain insights into how this technology works and deepen their understanding (Lee et al., 2022). Similarly, in the Bright and coworkers (2020) article (Bright et al., 2021), interviewed patients sought clear and concise information regarding PGx testing. In Allen and coworkers (2022) review, researchers concluded that patient counseling by healthcare experts is required to deal with literacy gaps, misconceptions, and prejudices (Allen et al., 2022). These findings collectively emphasize the importance of addressing the general public’s need for accessible and comprehensible information on PGx technology provided by well-trained healthcare professionals. The answer to this need could be provided by the use of artificial intelligence (AI), which is common in healthcare activities. Scientists have expanded and tested AI to PGx research and evaluation, and this could even be employed in patients’ information (Zack et al., 2025; Taherdoost and Ghofrani, 2024). Therefore, AI could be used to create open-source educational videos, literacy campaigns, or informational sessions to educate and counsel patients, aiming to make them familiar with the technology and less skeptical.

Our study has some limitations. First of all, the analysis was done in patients who consented to participate in the study, which could introduce a selection bias. Nevertheless, this is mitigated by the low consent rate, which would support the notion that people are not overly concerned about this. The second limitation is that our analysis only involved psychiatric patients, so we do not know if these findings will apply to other PGx indications (cardiovascular disease and/or oncology patients, chronic pain, etc.). Our findings are also focused on one country and are not developed in different countries and cultural settings, yet, both genders were equally represented along with group ages and educational level, making the study sample a consistent cohort.

To conclude, PGx testing is revolutionizing the clinical practice regarding drug prescription and management. Patients play a crucial role in the PGx process, serving as key stakeholders who should be actively involved both before and after receiving their PGx results. Valuing patients’ opinions and feedback is essential, as their input provides important insights to improve the overall PGx process. To ensure patients are well-informed and engaged, implementing a series of tailored activities is recommended. These may include actions such as individualized patient education, establishing collaborations with patients’ healthcare providers, and consistently updating patients’ PGx information over time. Such strategies will not only enhance patient understanding and involvement but also optimize the overall effectiveness of PGx testing in clinical practice.

## Data Availability

The original contributions presented in the study are included in the article/Supplementary Material, further inquiries can be directed to the corresponding author.

## Abbreviations

ADR, Adverse Drug Reaction; IQR, Interquartile Range; PREPARE, PREemptive Pharmacogenomic testing for preventing Adverse drug REactions; PM, Personalised Medicine; PGx, Pharmacogenomics; SD, Standard Deviation.
