# Supplementary material for: Assessing the views and opinions of psychiatric patients receiving genome-guided treatment within the scope of the PREPARE preemptive pharmacogenomics clinical study
Source: Front Pharmacol. 2025 Nov 21;16:1722339. doi: 10.3389/fphar.2025.1722339 (PMC12678367; doi:10.3389/fphar.2025.1722339)
Supplement: Supplementary file 1 [file Supplementaryfile1.docx]

**Supplementary Material**

**Supplementary Information.**

1. Supplemental file 1. U-PGx participants questionnaire
2. Supplemental file 2. Results of pairwise Spearman correlation of the questionnaire items.
3. Suppl. Table 2: Comparison of baseline patient characteristics between all the participants of the PGx-guided arm and the questionnaire patients.
4. Suppl. Table 3: Comparison of baseline patient characteristics between the participants of the PGx-guided arm and the questionnaire patients, focusing on patients with MD, BD and Schizophrenia (excluding Anxiety and Other patients). (=patients presented at Skokou et al.)
5. Suppl. Table 3: Cronbach’s alpha results
6. Suppl. Table 4: Impact of prior ADR experience on participants’ answers.
7. Suppl. Table 5: Impact of age on participants’ answers.
8. Suppl. Table 6. Impact of gender on participants’ answers.
9. Suppl. Table 7. Impact of level of education on participants’ answers.
10. Suppl. Table 8. Impact of psychiatric indication on participants’ answers.
11. Suppl. Figure 1. Participants' responses towards informedness and behaviour. This group of questions includes one more question (I have all the necessary information to understand how PGx works as a pharmacotherapy tool), to which all patients have responded “Agree” and is thus not visualised here.

**Supplemental file 1. U-PGx participants questionnaire**

[Questions to be answered before the administration of pharmacogenetic testing]

**A. Level of knowledge**

1. I am familiar with PGx. (1 = Disagree, 4 = Agree)
2. DNA can affect my response to medications. (1 = Disagree, 4 = Agree)

3. I am aware of the existence of PGx. (1 = Disagree, 4 = Agree)

**B. Perceived benefits**

4. PGx testing can reduce the occurrence and severity of adverse drug reactions (ADRs). (1 = Disagree, 4 = Agree)

5. PGx testing can help identify the most adequate medication for me. (1 = Disagree, 4 = Agree)

6. PGx testing can reduce the frequency of relapse occurrence. (1 = Disagree, 4 = Agree)

7. PGx testing can contribute to the reduction of total healthcare expenditures. (1 = Disagree, 4 = Agree)

8. I have noticed adverse drug reactions (ADRs) from a prescribed medication. (1 = Yes, 2 = No, 3 = I don’t remember)

9. Have you stopped taking a medication due to ineffectiveness? (1 = Yes, 2 = No, 3 = I don’t remember)

**C. General preferences**

10. Would you prefer to have PGx testing before being prescribed the index drug? (1 = Disagree, 4 = Agree)

11. Whose responsibility is to inform you and discuss your questions with based on your opinion? (1 = Physician, 2 = Researcher/Geneticist, 3 = Other)

[Questions to be answered upon determining the treatment scheme based on PGx results]

**D. Informedness and behaviour**

12. When prescribed a new medication, it is important to inform my physician about my PGx results by showing my PGx card. (1 = Disagree, 4 = Agree)

13. I have all the necessary information to understand how PGx works as a pharmacotherapy tool. (1 = Disagree, 4 = Agree)

14. I was properly informed about PGx before participating in this study. (1 = Disagree, 4 = Agree)

15. I believe that the benefits of PGx testing are clear. (1 = Disagree, 4 = Agree)

16. I trust more my physician who prescribes my medications. (1 = Yes, 2 = No, 3 = I don’t know)

17. I made changes to my medication therapy on my own after receiving my PGx results, without physician guidance. (1 = Not at all, 5 = Absolutely)

18. I made changes to my medication therapy after receiving my PGx results, based on my physician’s guidance. (1 = Not at all, 5 = Absolutely)

**E. Willingness to adopt**

19. After receiving my PGx results, I am more likely to follow the medication treatment recommended by my physician/pharmacist. (1 = Not at all, 5 = Absolutely)

20. I would recommend PGx testing to my children. (1 = Not at all, 5 = Absolutely)

21. I would recommend PGx testing to a friend or relative. (1 = Not at all, 5 = Absolutely)

22. I am interested in participating in a future PGx study. (1 = Not at all, 5 = Absolutely)

23. Would you like to learn more about PGx testing? (1 = Disagree, 4 = Agree)

**F. Barriers and concerns**

24. I am concerned that PGx testing will impact me financially. (1 = Disagree, 4 = Agree)

25. I am concerned about the confidentiality of my PGx testing results. (1 = Disagree, 4 = Agree)

26. I am concerned that my PGx information could be used in future research without my permission. (1 = Disagree, 4 = Agree)

**G. Overall satisfaction**

27. I am generally satisfied with my experience using PGx testing. (0 = Not at all, 10 = Absolutely)

28. PGx testing was helpful in making decisions about my health. (0 = Not at all, 10 = Absolutely)

**Suppl. Table 1:** Comparison of baseline patient characteristics between all the participants of PGx-guided arm and the questionnaire patients.

|  | **Entire PGx arm**, N = 646*^1^* | **Questionnaire PGx arm**, N = 201*^1^* | **p-value***^2^* |
| --- | --- | --- | --- |
| **Age (years)** | 48 (36, 57) | 48 (38, 58) | 0.7 |
| **Sex** |  |  | 0.5 |
| Female | 336 (52.01%) | 99 (49.25%) |  |
| Male | 310 (47.99%) | 102 (50.75%) |  |
| **BMI** |  |  | 0.7 |
| Healthy weight | 232 (35.91%) | 64 (31.84%) |  |
| Obese | 153 (23.68%) | 52 (25.87%) |  |
| Overweight | 249 (38.54%) | 81 (40.30%) |  |
| Underweight | 12 (1.86%) | 4 (1.99%) |  |
| **Smoking Status** |  |  | 0.7 |
| Current | 357 (55.26%) | 114 (56.72%) |  |
| Ex-smoker | 65 (10.06%) | 16 (7.96%) |  |
| Non-smoker | 224 (34.67%) | 71 (35.32%) |  |
| **Alcohol consumption (units/week)** |  |  | 0.5 |
| <1 | 435 (67.34%) | 146 (72.64%) |  |
| >50 | 10 (1.55%) | 2 (1.00%) |  |
| 1-5 | 131 (20.28%) | 37 (18.41%) |  |
| 15-21 | 14 (2.17%) | 6 (2.99%) |  |
| 22-49 | 8 (1.24%) | 1 (0.50%) |  |
| 6-14 | 48 (7.43%) | 9 (4.48%) |  |
| **Indication category** |  |  | 0.4 |
| Anxiety Disorders | 42 (6.50%) | 7 (3.48%) |  |
| Bipolar Disorder | 139 (21.52%) | 40 (19.90%) |  |
| Major Depression | 261 (40.40%) | 89 (44.28%) |  |
| Other | 57 (8.82%) | 15 (7.46%) |  |
| Schizophrenia | 147 (22.76%) | 50 (24.88%) |  |
| **CYP2D6 Metaboliser Status** |  |  | 0.7 |
| Extensive/Normal | 362 (56.56%) | 114 (57.87%) |  |
| Intermediate | 211 (32.97%) | 58 (29.44%) |  |
| Poor | 48 (7.50%) | 19 (9.64%) |  |
| Ultrarapid | 19 (2.97%) | 6 (3.05%) |  |
| Unknown | 6 | 4 |  |
| **CYP2C19 Metaboliser Status** |  |  | 0.7 |
| Extensive/Normal | 364 (63.97%) | 114 (66.67%) |  |
| Intermediate | 148 (26.01%) | 45 (26.32%) |  |
| Poor | 38 (6.68%) | 8 (4.68%) |  |
| Ultrarapid | 19 (3.34%) | 4 (2.34%) |  |
| Unknown | 77 | 30 |  |
| **Actionable patients (n)** | 163 (25.23%) | 51 (25.37%) | >0.9 |
| **Baseline Utility (N1)** | 0.59 (0.40, 0.70) | 0.55 (0.40, 0.70) | 0.3 |
| Unknown | 99 | 22 |  |
| **Index Drug** |  |  | 0.5 |
| Amitriptyline | 16 (2.48%) | 1 (0.50%) |  |
| Aripiprazole | 116 (17.96%) | 41 (20.40%) |  |
| Carbamazepine | 4 (0.62%) | 0 (0.00%) |  |
| Citalopram | 83 (12.85%) | 29 (14.43%) |  |
| Clomipramine | 11 (1.70%) | 2 (1.00%) |  |
| Doxepine | 1 (0.15%) | 1 (0.50%) |  |
| Escitalopram | 101 (15.63%) | 22 (10.95%) |  |
| Haloperidol | 71 (10.99%) | 22 (10.95%) |  |
| Paroxetine | 28 (4.33%) | 8 (3.98%) |  |
| Pimozide | 1 (0.15%) | 0 (0.00%) |  |
| Sertraline | 115 (17.80%) | 34 (16.92%) |  |
| Venlafaxine | 84 (13.00%) | 36 (17.91%) |  |
| Zuclopenthixol | 15 (2.32%) | 5 (2.49%) |  |
| **Category of Index Drug** |  |  | 0.6 |
| Antidepressant | 439 (67.96%) | 133 (66.17%) |  |
| Antipsychotic | 203 (31.42%) | 68 (33.83%) |  |
| Mood stabilizer | 4 (0.62%) | 0 (0.00%) |  |
| *^1^* Median (IQR); n (%) *^2^* Wilcoxon rank sum test; Pearson's Chi-squared test; Fisher's exact test | | | |

**Suppl. Table 2:** Comparison of baseline patient characteristics between the participants of PGx-guided arm and the questionnaire patients, focusing on patients with MD, BD and Schizophrenia (excluding Anxiety and Other patients). (=patients presented at Skokou and coworkers, (2024)).

|  | **Entire PGx arm**,  N = 547*^1^* | **Questionnaire PGx arm**,  N = 179*^1^* | **p-value***^2^* |
| --- | --- | --- | --- |
| **Age (years)** | 49 (39, 58) | 49 (40, 58) | >0.9 |
| **Sex** |  |  | 0.6 |
| Female | 285 (52.10%) | 89 (49.72%) |  |
| Male | 262 (47.90%) | 90 (50.28%) |  |
| **BMI** |  |  | 0.7 |
| Healthy weight | 177 (32.36%) | 51 (28.49%) |  |
| Obese | 139 (25.41%) | 50 (27.93%) |  |
| Overweight | 224 (40.95%) | 75 (41.90%) |  |
| Underweight | 7 (1.28%) | 3 (1.68%) |  |
| **Smoking Status** |  |  | 0.6 |
| Current | 316 (57.77%) | 104 (58.10%) |  |
| Ex-smoker | 50 (9.14%) | 12 (6.70%) |  |
| Non-smoker | 181 (33.09%) | 63 (35.20%) |  |
| **Alcohol consumption (units/week)** |  |  | 0.3 |
| <1 | 380 (69.47%) | 132 (73.74%) |  |
| >50 | 5 (0.91%) | 0 (0.00%) |  |
| 1-5 | 103 (18.83%) | 35 (19.55%) |  |
| 15-21 | 10 (1.83%) | 4 (2.23%) |  |
| 22-49 | 7 (1.28%) | 0 (0.00%) |  |
| 6-14 | 42 (7.68%) | 8 (4.47%) |  |
| **Indication category** |  |  | 0.7 |
| Bipolar Disorder | 139 (25.41%) | 40 (22.35%) |  |
| Major Depression | 261 (47.71%) | 89 (49.72%) |  |
| Schizophrenia | 147 (26.87%) | 50 (27.93%) |  |
| **CYP2D6 Metaboliser Status** |  |  | 0.4 |
| Extensive/Normal | 306 (56.46%) | 103 (58.86%) |  |
| Intermediate | 175 (32.29%) | 47 (26.86%) |  |
| Poor | 43 (7.93%) | 19 (10.86%) |  |
| Ultrarapid | 18 (3.32%) | 6 (3.43%) |  |
| Unknown | 5 | 4 |  |
| **CYP2C19 Metaboliser Status** |  |  | 0.5 |
| Extensive/Normal | 307 (64.09%) | 103 (68.21%) |  |
| Intermediate | 125 (26.10%) | 39 (25.83%) |  |
| Poor | 29 (6.05%) | 5 (3.31%) |  |
| Ultrarapid | 18 (3.76%) | 4 (2.65%) |  |
| Unknown | 68 | 28 |  |
| **Actionable patients (n)** | 136 (24.86%) | 43 (24.02%) | 0.8 |
| **Baseline Utility (N1)** | 0.59 (0.40, 0.70) | 0.55 (0.40, 0.70) | 0.3 |
| **Index Drug** |  |  | 0.6 |
| Amitriptyline | 16 (2.93%) | 1 (0.56%) |  |
| Aripiprazole | 110 (20.11%) | 39 (21.79%) |  |
| Carbamazepine | 3 (0.55%) | 0 (0.00%) |  |
| Citalopram | 71 (12.98%) | 27 (15.08%) |  |
| Clomipramine | 6 (1.10%) | 2 (1.12%) |  |
| Doxepine | 1 (0.18%) | 1 (0.56%) |  |
| Escitalopram | 85 (15.54%) | 21 (11.73%) |  |
| Haloperidol | 64 (11.70%) | 19 (10.61%) |  |
| Paroxetine | 16 (2.93%) | 5 (2.79%) |  |
| Pimozide | 1 (0.18%) | 0 (0.00%) |  |
| Sertraline | 86 (15.72%) | 26 (14.53%) |  |
| Venlafaxine | 75 (13.71%) | 34 (18.99%) |  |
| Zuclopenthixol | 13 (2.38%) | 4 (2.23%) |  |
| **Category of Index Drug** |  |  | >0.9 |
| Antidepressant | 356 (65.08%) | 117 (65.36%) |  |
| Antipsychotic | 188 (34.37%) | 62 (34.64%) |  |
| Mood stabilizer | 3 (0.55%) | 0 (0.00%) |  |
| *^1^* Median (IQR); n (%) *^2^* Wilcoxon rank sum test; Pearson's Chi-squared test; Fisher's exact test | | | |

**Suppl. Table 3:** Cronbach’s alpha results

|  | Alpha | Internal Consistency |
| --- | --- | --- |
| Barriers and concerns | 0.96 | Excellent |
| Level of knowledge | 0.85 | Good |
| Overall satisfaction | 0.87 | Good |
| Perceived benefits | 0.55 | Poor |
| Informedness and Behaviour | 0.26 | Unacceptable |
| General preferences | 0.039 | Unacceptable |
| Willingness to adopt | 0.7 | Acceptable |
| Overall questionnaire | 0.75 | Acceptable |

**Suppl. Table 4:** Impact of prior ADR experience on participants’ answers. Note, two of the 28 questions in the questionnaire were not included in these comparisons due to zero variance in the provided responses. See Data Analysis section in the main manuscript. As a result, the total questions presented here are 26. The p-value and adjusted p-value refer to the comparison between the individual groups (Yes, No) and not between the groups and overall.

|  | | **Overall**, N = 201*^1^* | | **Yes**, N = 106*^1^* | | **No**, N = 95*^1^* | **p-value***^2^* | | **q-value***^3^* | | | | |  |  |
| --- | --- | --- | --- | --- | --- | --- | --- | --- | --- | --- | --- | --- | --- | --- | --- |
| ***^2a^* I am familiar with PGx. (1 = Disagree, 4 = Agree)** | | | | | |  | 0.2 | | 0.5 | | | | |  |  |
| 1 | | 168 (83.58%) | 89 (83.96%) | | 79 (83.16%) | | |  | |  | | |  | | |
| 2 | | 4 (1.99%) | 0 (0.00%) | | 4 (4.21%) | | |  | |  | | |  | | |
| 3 | | 2 (1.00%) | 1 (0.94%) | | 1 (1.05%) | | |  | |  | | |  | | |
| 4 | | 27 (13.43%) | 16 (15.09%) | | 11 (11.58%) | | |  | |  | | |  | | |
| ***^2a^* DNA can affect my response to medications. (1 = Disagree, 4 = Agree)** | | | | | |  | 0.4 | | 0.8 | | | | |  |  |
| 1 | | 9 (4.48%) | | 7 (6.60%) | | 2 (2.11%) |  | |  | |  | | |  |  |
| 2 | | 2 (1.00%) | | 1 (0.94%) | | 1 (1.05%) |  | |  | |  | | |  |  |
| 3 | | 5 (2.49%) | | 3 (2.83%) | | 2 (2.11%) |  | |  | |  | | |  |  |
| 4 | | 185 (92.04%) | | 95 (89.62%) | | 90 (94.74%) |  | |  | |  | | |  |  |
| ***^2a^* I am aware of the existence of PGx. (1 = Disagree, 4 = Agree)** | | | | | |  | 0.6 | | 0.8 | | | | |  |  |
| 1 | | 149 (74.13%) | | 79 (74.53%) | | 70 (73.68%) |  | |  | | | | |  |  |
| 2 | | 20 (9.95%) | | 8 (7.55%) | | 12 (12.63%) |  | |  | | | | |  |  |
| 3 | | 10 (4.98%) | | 6 (5.66%) | | 4 (4.21%) |  | |  | | | | |  |  |
| 4 | | 22 (10.95%) | | 13 (12.26%) | | 9 (9.47%) |  | |  | | | | |  |  |
| ***^2a^* PGx testing can reduce the occurrence and severity of adverse drug reactions (ADRs). (1 = Disagree, 4 = Agree)** | | | | | |  | 0.8 | | 0.9 | | | | |  |  |
| 1 | | 1 (0.50%) | | 0 (0.00%) | | 1 (1.05%) |  | |  | | | | |  |  |
| 3 | | 3 (1.49%) | | 2 (1.89%) | | 1 (1.05%) |  | |  | | | | |  |  |
| 4 | | 197 (98.01%) | | 104 (98.11%) | | 93 (97.89%) |  | |  | | | | |  |  |
| ***^2b^* PGx testing can help identify the most adequate medication for me.** | | | | | |  | 0.12 | | 0.4 | | | | |  |  |
| **(1 = Disagree, 4 = Agree)** | | | | | |  |  |  |  |  |  |  |  |  |  |
| 3 | | 17 (8.46%) | | 12 (11.32%) | | 5 (5.26%) |  | |  | | | | |  |  |
| 4 | | 184 (91.54%) | | 94 (88.68%) | | 90 (94.74%) |  | |  | | | | |  |  |
| ***^2a^* PGx testing can reduce the frequency of relapse occurrence. (1 = Disagree, 4 = Agree)** | | | | | |  | 0.4 | | 0.8 | | | | |  |  |
| 2 | | 2 (1.00%) | | 0 (0.00%) | | 2 (2.11%) |  | |  | | | | |  |  |
| 3 | | 59 (29.35%) | | 30 (28.30%) | | 29 (30.53%) |  | |  | | | | |  |  |
| 4 | | 140 (69.65%) | | 76 (71.70%) | | 64 (67.37%) |  | |  | | | | |  |  |
| ***^2a^* PGx testing can contribute to the reduction of total healthcare expenditures. (1 = Disagree, 4 = Agree)** | | | | | |  | 0.6 | | 0.8 | | | | |  |  |
| 1 | | 1 (0.50%) | | 0 (0.00%) | | 1 (1.05%) |  | |  | | | | |  |  |
| 2 | | 23 (11.44%) | | 10 (9.43%) | | 13 (13.68%) |  | |  | | | | |  |  |
| 3 | | 82 (40.80%) | | 45 (42.45%) | | 37 (38.95%) |  | |  | | | | |  |  |
| 4 | | 95 (47.26%) | | 51 (48.11%) | | 44 (46.32%) |  | |  | | | | |  |  |
| ***^2a^* Would you prefer to have PGx testing before being prescribed the index drug? (1 = Disagree, 4 = Agree)** | | | | | |  | >0.9 | | >0.9 | | | | |  |  |
| 3 | | 2 (1.00%) | | 1 (0.94%) | | 1 (1.05%) |  | |  | | | | |  |  |
| 4 | | 199 (99.00%) | | 105 (99.06%) | | 94 (98.95%) |  | |  | | | | |  |  |
| ***^2a^* Would you like to learn more about PGx testing? (1 = Disagree, 4 = Agree)** | | | | | |  | 0.6 | | 0.8 | | | | |  |  |
| 1 | | 29 (14.43%) | | 14 (13.21%) | | 15 (15.79%) |  | |  | | | | |  |  |
| 2 | | 5 (2.49%) | | 4 (3.77%) | | 1 (1.05%) |  | |  | | | | |  |  |
| 3 | | 4 (1.99%) | | 2 (1.89%) | | 2 (2.11%) |  | |  | | | | |  |  |
| 4 | | 163 (81.09%) | | 86 (81.13%) | | 77 (81.05%) |  | |  | | | | |  |  |
| ***^2a^* Whose responsibility is to inform you and discuss your questions with based on your opinion?** | | | | | |  | 0.6 | | 0.8 | | | | |  |  |
| Physician | | 138 (68.66%) | | 76 (71.70%) | | 62 (65.26%) |  | |  | | | | |  |  |
| Researcher/Geneticist | | 59 (29.35%) | | 28 (26.42%) | | 31 (32.63%) |  | |  | | | | |  |  |
| Other | | 4 (1.99%) | | 2 (1.89%) | | 2 (2.11%) |  | |  | | | | |  |  |
| ***^2a^* When prescribed a new medication, it is important to inform my physician about my PGx results by showing my PGx card. (1 = Disagree, 4 = Agree)** | | | | | |  | 0.4 | | 0.8 | | | | |  |  |
| 1 | | 2 (1.00%) | | 2 (1.89%) | | 0 (0.00%) |  | |  | | | | |  |  |
| 2 | | 1 (0.50%) | | 0 (0.00%) | | 1 (1.05%) |  | |  | | | | |  |  |
| 4 | | 198 (98.51%) | | 104 (98.11%) | | 94 (98.95%) |  | |  | | | | |  |  |
| ***^2a^* I was properly informed about PGx before participating in this study. (1 = Disagree, 4 = Agree)** | | | | | |  | 0.5 | | 0.8 | | | | |  |  |
| 3 | | 1 (0.50%) | | 0 (0.00%) | | 1 (1.05%) |  | |  | | | | |  |  |
| 4 | | 200 (99.50%) | | 106 (100.00%) | | 94 (98.95%) |  | |  | | | | |  |  |
| ***^2b^* I believe that the benefits of PGx testing are clear. (1 = Disagree, 4 = Agree)** | | | | | |  | 0.6 | | 0.8 | | | | |  |  |
| 3 | | 43 (21.39%) | | 24 (22.64%) | | 19 (20.00%) |  | |  | | | | |  |  |
| 4 | | 158 (78.61%) | | 82 (77.36%) | | 76 (80.00%) |  | |  | | | | |  |  |
| ***^2b^* I trust more my physician who prescribes my medications.** | | | | | |  | >0.9 | | >0.9 | | | | |  |  |
| I don't know | | 32 (15.92%) | | 16 (15.09%) | | 16 (16.84%) |  | |  | | | | |  |  |
| No | | 25 (12.44%) | | 14 (13.21%) | | 11 (11.58%) |  | |  | | | | |  |  |
| Yes | | 144 (71.64%) | | 76 (71.70%) | | 68 (71.58%) |  | |  | | | | |  |  |
| ***^2a^* After receiving my PGx results, I am more likely to follow the medication treatment recommended by my physician/pharmacist. (1 = Not at all, 5 = Absolutely)** | | | | | |  | 0.3 | | 0.7 | | | | |  |  |
| 2 | | 4 (1.99%) | | 1 (0.94%) | | 3 (3.16%) |  | |  | | | | |  |  |
| 3 | | 15 (7.46%) | | 11 (10.38%) | | 4 (4.21%) |  | |  | | | | |  |  |
| 4 | | 77 (38.31%) | | 39 (36.79%) | | 38 (40.00%) |  | |  | | | | |  |  |
| 5 | | 105 (52.24%) | | 55 (51.89%) | | 50 (52.63%) |  | |  | | | | |  |  |
| ***^2b^* I would recommend PGx testing to my children. (1 = Not at all, 5 = Absolutely)** | | | | | |  | 0.7 | | 0.8 | | | | |  |  |
| 3 | | 11 (5.47%) | | 5 (4.72%) | | 6 (6.32%) |  | |  | | | | |  |  |
| 4 | | 86 (42.79%) | | 48 (45.28%) | | 38 (40.00%) |  | |  | | | | |  |  |
| 5 | | 104 (51.74%) | | 53 (50.00%) | | 51 (53.68%) |  | |  | | | | |  |  |
| ***^2a^* I would recommend PGx testing to a friend or relative. (1 = Not at all, 5 = Absolutely)** | | | | | |  | 0.7 | | 0.8 | | | | |  |  |
| 1 | | 1 (0.50%) | | 1 (0.94%) | | 0 (0.00%) |  | |  | | | | |  |  |
| 3 | | 11 (5.47%) | | 5 (4.72%) | | 6 (6.32%) |  | |  | | | | |  |  |
| 4 | | 87 (43.28%) | | 49 (46.23%) | | 38 (40.00%) |  | |  | | | | |  |  |
| 5 | | 102 (50.75%) | | 51 (48.11%) | | 51 (53.68%) |  | |  | | | | |  |  |
| ***^2b^* I am interested in participating in a future PGx study. (1 = Not at all, 5 = Absolutely)** | | | | | |  | 0.2 | | 0.6 | | | | |  |  |
| 1 | | 15 (7.46%) | | 6 (5.66%) | | 9 (9.47%) |  | |  | | | | |  |  |
| 2 | | 18 (8.96%) | | 11 (10.38%) | | 7 (7.37%) |  | |  | | | | |  |  |
| 3 | | 48 (23.88%) | | 24 (22.64%) | | 24 (25.26%) |  | |  | | | | |  |  |
| 4 | | 60 (29.85%) | | 38 (35.85%) | | 22 (23.16%) |  | |  | | | | |  |  |
| 5 | | 60 (29.85%) | | 27 (25.47%) | | 33 (34.74%) |  | |  | | | | |  |  |
| ***^2a^* I made changes to my medication therapy on my own after receiving my PGx results, without physician guidance. (1 = Not at all, 5 = Absolutely)** | | | | | |  | **0.041** | | **0.3** | | | | |  |  |
| 1 | | 195 (97.01%) | | 101 (95.28%) | | 94 (98.95%) |  | |  | | | | |  |  |
| 2 | | 1 (0.50%) | | 0 (0.00%) | | 1 (1.05%) |  | |  | | | | |  |  |
| 3 | | 5 (2.49%) | | 5 (4.72%) | | 0 (0.00%) |  | |  | | | | |  |  |
| ***^2a^* I made changes to my medication therapy after receiving my PGx results, based on my physician’s guidance. (1 = Not at all, 5 = Absolutely)** | | | | | |  | **<0.001** | | **0.003** | | | | |  |  |
| 1 | | 105 (52.24%) | | 42 (39.62%) | | 63 (66.32%) |  | |  | | | | |  |  |
| 2 | | 1 (0.50%) | | 0 (0.00%) | | 1 (1.05%) |  | |  | | | | |  |  |
| 3 | | 18 (8.96%) | | 13 (12.26%) | | 5 (5.26%) |  | |  | | | | |  |  |
| 4 | | 6 (2.99%) | | 6 (5.66%) | | 0 (0.00%) |  | |  | | | | |  |  |
| 5 | | 71 (35.32%) | | 45 (42.45%) | | 26 (27.37%) |  | |  | | | | |  |  |
| ***^2b^* Have you stopped taking a medication due to ineffectiveness? (1 = Yes, 2 = No, 3 = I don’t remember)** | | | | | |  | **<0.001** | | **<0.001** | | | | |  |  |
| Yes | | 103 (51.24%) | | 88 (83.02%) | | 15 (15.79%) |  | |  | | | | |  |  |
| No | | 98 (48.76%) | | 18 (16.98%) | | 80 (84.21%) |  | |  | | | | |  |  |
| ***^2a^* I am concerned about the confidentiality of my PGx testing results. (1 = Disagree, 4 = Agree)** | | | | | |  | **0.039** | | **0.3** | | | | |  |  |
| 1 | | 186 (92.54%) | | 95 (89.62%) | | 91 (95.79%) |  | |  | | | | |  |  |
| 2 | | 14 (6.97%) | | 11 (10.38%) | | 3 (3.16%) |  | |  | | | | |  |  |
| 3 | | 1 (0.50%) | | 0 (0.00%) | | 1 (1.05%) |  | |  | | | | |  |  |
| ***^2a^* I am concerned that my PGx information could be used in future research without my permission. (1 = Disagree, 4 = Agree)** | | | | | |  | 0.12 | | 0.4 | | | | |  |  |
| 1 | | 186 (92.54%) | | 95 (89.62%) | | 91 (95.79%) |  | |  | | | | |  |  |
| 2 | | 13 (6.47%) | | 10 (9.43%) | | 3 (3.16%) |  | |  | | | | |  |  |
| 3 | | 2 (1.00%) | | 1 (0.94%) | | 1 (1.05%) |  | |  | | | | |  |  |
| ***^2a^* I am generally satisfied with my experience using PGx testing. (0 = Not at all, 10 = Absolutely)** | | | | | |  | 0.11 | | 0.4 | | | | |  |  |
| 6 | | 3 (1.49%) | | 0 (0.00%) | | 3 (3.16%) |  | |  | | | | |  |  |
| 7 | | 7 (3.48%) | | 6 (5.66%) | | 1 (1.05%) |  | |  | | | | |  |  |
| 8 | | 34 (16.92%) | | 20 (18.87%) | | 14 (14.74%) |  | |  | | | | |  |  |
| 9 | | 60 (29.85%) | | 33 (31.13%) | | 27 (28.42%) |  | |  | | | | |  |  |
| 10 | | 97 (48.26%) | | 47 (44.34%) | | 50 (52.63%) |  | |  | | | | |  |  |
| ***^2a^* PGx testing was helpful in making decisions about my health. (0 = Not at all, 10 = Absolutely)** | | | | | |  | 0.5 | | 0.8 | | | | |  |  |
| 5 | | 1 (0.50%) | | 0 (0.00%) | | 1 (1.05%) |  | |  | | | | |  |  |
| 7 | | 16 (7.96%) | | 8 (7.55%) | | 8 (8.42%) |  | |  | | | | |  |  |
| 8 | | 20 (9.95%) | | 11 (10.38%) | | 9 (9.47%) |  | |  | | | | |  |  |
| 9 | | 52 (25.87%) | | 32 (30.19%) | | 20 (21.05%) |  | |  | | | | |  |  |
| 10 | | 112 (55.72%) | | 55 (51.89%) | | 57 (60.00%) |  | |  | | | | |  |  |
|  | *^1^* n (%),*^2a^* Fisher's exact test; *^2b^* Pearson's Chi-squared test, *^3^* Benjamini & Hochberg correction for multiple testing | | | | | | | | | | |  | | |  |

**Suppl. Table 5:** Impact of age on participants’ answers. Note, two of the 28 questions in the questionnaire were not included in these comparisons due to zero variance in the provided responses. See Data Analysis section in the main manuscript. As a result, the total questions presented here are 26. The p-value and adjusted p-value refer to the comparison between the individual age groups and not between the groups and overall.

|  | | **Overall**, | **18-34**, | **35-44**, | | **45-54**, | | **55-64**, | | **65+**, | | | **p-value***^2^* | | | **q-value***^3^* | | |  |  |  |
| --- | --- | --- | --- | --- | --- | --- | --- | --- | --- | --- | --- | --- | --- | --- | --- | --- | --- | --- | --- | --- | --- |
|  |  | N = 201*^1^* | N = 36*^1^* | N = 40*^1^* | | N = 60*^1^* | | N = 48*^1^* | | N = 17*^1^* | | |  |  |  |  |  |  |  |  |  |
| ***^2a^* I am familiar with PGx. (1 = Disagree, 4 = Agree)** | | | | | | | | | | | |  | | | 0.14 | | | 0.6 | | | |
| 1 | | 168 (83.58%) | 26 (72.22%) | | 35 (87.50%) | | 48 (80.00%) | | 44 (91.67%) | | 15 (88.24%) | | |  | | |  | | |  |  |
| 2 | | 4 (1.99%) | 1 (2.78%) | | 1 (2.50%) | | 2 (3.33%) | | 0 (0.00%) | | 0 (0.00%) | | |  | | |  | | |  |  |
| 3 | | 2 (1.00%) | 0 (0.00%) | | 0 (0.00%) | | 0 (0.00%) | | 1 (2.08%) | | 1 (5.88%) | | |  | | |  | | |  |  |
| 4 | | 27 (13.43%) | 9 (25.00%) | | 4 (10.00%) | | 10 (16.67%) | | 3 (6.25%) | | 1 (5.88%) | | |  | | |  | | |  |  |
| ***^2a^* DNA can affect my response to medications. (1 = Disagree, 4 = Agree)** | | | | | | | | | | | |  | | | >0.9 | | | >0.9 | | | |
| 1 | | 9 (4.48%) | 1 (2.78%) | | 2 (5.00%) | | 4 (6.67%) | | 1 (2.08%) | | 1 (5.88%) | | |  | | |  | | |  |  |
| 2 | | 2 (1.00%) | 1 (2.78%) | | 0 (0.00%) | | 1 (1.67%) | | 0 (0.00%) | | 0 (0.00%) | | |  | | |  | | |  |  |
| 3 | | 5 (2.49%) | 1 (2.78%) | | 1 (2.50%) | | 1 (1.67%) | | 1 (2.08%) | | 1 (5.88%) | | |  | | |  | | |  |  |
| 4 | | 185 (92.04%) | 33 (91.67%) | | 37 (92.50%) | | 54 (90.00%) | | 46 (95.83%) | | 15 (88.24%) | | |  | | |  | | |  |  |
| ***^2a^* I am aware of the existence of PGx. (1 = Disagree, 4 = Agree)** | | | | | | | | | | | |  | | | **0.030** | | | **0.2** | | | |
| 1 | | 149 (74.13%) | 21 (58.33%) | | 30 (75.00%) | | 41 (68.33%) | | 44 (91.67%) | | 13 (76.47%) | | |  | | |  | | |  |  |
| 2 | | 20 (9.95%) | 4 (11.11%) | | 6 (15.00%) | | 8 (13.33%) | | 0 (0.00%) | | 2 (11.76%) | | |  | | |  | | |  |  |
| 3 | | 10 (4.98%) | 3 (8.33%) | | 2 (5.00%) | | 3 (5.00%) | | 1 (2.08%) | | 1 (5.88%) | | |  | | |  | | |  |  |
| 4 | | 22 (10.95%) | 8 (22.22%) | | 2 (5.00%) | | 8 (13.33%) | | 3 (6.25%) | | 1 (5.88%) | | |  | | |  | | |  |  |
| ***^2a^* PGx testing can reduce the occurrence and severity of adverse drug reactions (ADRs). (1 = Disagree, 4 = Agree)** | | | | | | | | | | | |  | | | 0.9 | | | >0.9 | | | |
| 1 | | 1 (0.50%) | 1 (2.78%) | | 0 (0.00%) | | 0 (0.00%) | | 0 (0.00%) | | 0 (0.00%) | | |  | | |  | | |  |  |
| 3 | | 3 (1.49%) | 0 (0.00%) | | 1 (2.50%) | | 1 (1.67%) | | 1 (2.08%) | | 0 (0.00%) | | |  | | |  | | |  |  |
| 4 | | 197 (98.01%) | 35 (97.22%) | | 39 (97.50%) | | 59 (98.33%) | | 47 (97.92%) | | 17 (100.00%) | | |  | | |  | | |  |  |
| ***^2a^* PGx testing can help identify the most adequate medication for me. (1 = Disagree, 4 = Agree)** | | | | | | | | | | | |  | | | 0.8 | | | >0.9 | | | |
| 3 | | 17 (8.46%) | 2 (5.56%) | | 4 (10.00%) | | 7 (11.67%) | | 3 (6.25%) | | 1 (5.88%) | | |  | | |  | | |  |  |
| 4 | | 184 (91.54%) | 34 (94.44%) | | 36 (90.00%) | | 53 (88.33%) | | 45 (93.75%) | | 16 (94.12%) | | |  | | |  | | |  |  |
| ***^2a^* PGx testing can reduce the frequency of relapse occurrence. (1 = Disagree, 4 = Agree)** | | | | | | | | | | | |  | | | >0.9 | | | >0.9 | | | |
| 2 | | 2 (1.00%) | 0 (0.00%) | | 1 (2.50%) | | 0 (0.00%) | | 1 (2.08%) | | 0 (0.00%) | | |  | | |  | | |  |  |
| 3 | | 59 (29.35%) | 12 (33.33%) | | 10 (25.00%) | | 19 (31.67%) | | 14 (29.17%) | | 4 (23.53%) | | |  | | |  | | |  |  |
| 4 | | 140 (69.65%) | 24 (66.67%) | | 29 (72.50%) | | 41 (68.33%) | | 33 (68.75%) | | 13 (76.47%) | | |  | | |  | | |  |  |
| ***^2a^* PGx testing can contribute to the reduction of total healthcare expenditures. (1 = Disagree, 4 = Agree)** | | | | | | | | | | | |  | | | >0.9 | | | >0.9 | | | |
| 1 | | 1 (0.50%) | 0 (0.00%) | | 0 (0.00%) | | 1 (1.67%) | | 0 (0.00%) | | 0 (0.00%) | | |  | | |  | | |  |  |
| 2 | | 23 (11.44%) | 6 (16.67%) | | 2 (5.00%) | | 7 (11.67%) | | 6 (12.50%) | | 2 (11.76%) | | |  | | |  | | |  |  |
| 3 | | 82 (40.80%) | 13 (36.11%) | | 19 (47.50%) | | 25 (41.67%) | | 18 (37.50%) | | 7 (41.18%) | | |  | | |  | | |  |  |
| 4 | | 95 (47.26%) | 17 (47.22%) | | 19 (47.50%) | | 27 (45.00%) | | 24 (50.00%) | | 8 (47.06%) | | |  | | |  | | |  |  |
| ***^2a^* Would you prefer to have PGx testing before being prescribed the index drug? (1 = Disagree, 4 = Agree)** | | | | | | | | | | | |  | | | 0.6 | | | >0.9 | | | |
| 3 | | 2 (1.00%) | 0 (0.00%) | | 1 (2.50%) | | 0 (0.00%) | | 1 (2.08%) | | 0 (0.00%) | | |  | | |  | | |  |  |
| 4 | | 199 (99.00%) | 36 (100.00%) | | 39 (97.50%) | | 60 (100.00%) | | 47 (97.92%) | | 17 (100.00%) | | |  | | |  | | |  |  |
| ***^2a^* Would you like to learn more about PGx testing? (1 = Disagree, 4 = Agree)** | | | | | | | | | | | |  | | | 0.4 | | | 0.9 | | | |
| 1 | | 29 (14.43%) | 2 (5.56%) | | 6 (15.00%) | | 11 (18.33%) | | 7 (14.58%) | | 3 (17.65%) | | |  | | |  | | |  |  |
| 2 | | 5 (2.49%) | 0 (0.00%) | | 1 (2.50%) | | 1 (1.67%) | | 2 (4.17%) | | 1 (5.88%) | | |  | | |  | | |  |  |
| 3 | | 4 (1.99%) | 0 (0.00%) | | 1 (2.50%) | | 0 (0.00%) | | 3 (6.25%) | | 0 (0.00%) | | |  | | |  | | |  |  |
| 4 | | 163 (81.09%) | 34 (94.44%) | | 32 (80.00%) | | 48 (80.00%) | | 36 (75.00%) | | 13 (76.47%) | | |  | | |  | | |  |  |
| ***^2a^* Whose responsibility is to inform you and discuss your questions with based on your opinion?** | | | | | | | | | | | |  | | | **0.004** | | | **0.10** | | | |
| Physician | | 138 (68.66%) | 16 (44.44%) | | 28 (70.00%) | | 40 (66.67%) | | 38 (79.17%) | | 16 (94.12%) | | |  | | |  | | |  |  |
| Researcher/Geneticist | | 59 (29.35%) | 20 (55.56%) | | 11 (27.50%) | | 18 (30.00%) | | 9 (18.75%) | | 1 (5.88%) | | |  | | |  | | |  |  |
| Other | | 4 (1.99%) | 0 (0.00%) | | 1 (2.50%) | | 2 (3.33%) | | 1 (2.08%) | | 0 (0.00%) | | |  | | |  | | |  |  |
| ***^2a^* When prescribed a new medication, it is important to inform my physician about my PGx results by showing my PGx card. (1 = Disagree, 4 = Agree)** | | | | | | | | | | | |  | | | 0.4 | | | 0.9 | | | |
| 1 | | 2 (1.00%) | 0 (0.00%) | | 0 (0.00%) | | 2 (3.33%) | | 0 (0.00%) | | 0 (0.00%) | | |  | | |  | | |  |  |
| 2 | | 1 (0.50%) | 1 (2.78%) | | 0 (0.00%) | | 0 (0.00%) | | 0 (0.00%) | | 0 (0.00%) | | |  | | |  | | |  |  |
| 4 | | 198 (98.51%) | 35 (97.22%) | | 40 (100.00%) | | 58 (96.67%) | | 48 (100.00%) | | 17 (100.00%) | | |  | | |  | | |  |  |
| ***^2a^* I was properly informed about PGx before participating in this study. (1 = Disagree, 4 = Agree)** | | | | | | | | | | | |  | | | >0.9 | | | >0.9 | | | |
| 3 | | 1 (0.50%) | 0 (0.00%) | | 0 (0.00%) | | 1 (1.67%) | | 0 (0.00%) | | 0 (0.00%) | | |  | | |  | | |  |  |
| 4 | | 200 (99.50%) | 36 (100.00%) | | 40 (100.00%) | | 59 (98.33%) | | 48 (100.00%) | | 17 (100.00%) | | |  | | |  | | |  |  |
| ***^2a^* I believe that the benefits of PGx testing are clear. (1 = Disagree, 4 = Agree)** | | | | | | | | | | | |  | | | 0.4 | | | 0.9 | | | |
| 3 | | 43 (21.39%) | 9 (25.00%) | | 11 (27.50%) | | 11 (18.33%) | | 11 (22.92%) | | 1 (5.88%) | | |  | | |  | | |  |  |
| 4 | | 158 (78.61%) | 27 (75.00%) | | 29 (72.50%) | | 49 (81.67%) | | 37 (77.08%) | | 16 (94.12%) | | |  | | |  | | |  |  |
| ***^2a^* I trust more my physician who prescribes my medications.** | | | | | | | | | | | |  | | | 0.9 | | | >0.9 | | | |
| I don't know | | 32 (15.92%) | 8 (22.22%) | | 6 (15.00%) | | 9 (15.00%) | | 7 (14.58%) | | 2 (11.76%) | | |  | | |  | | |  |  |
| No | | 25 (12.44%) | 2 (5.56%) | | 7 (17.50%) | | 7 (11.67%) | | 6 (12.50%) | | 3 (17.65%) | | |  | | |  | | |  |  |
| Yes | | 144 (71.64%) | 26 (72.22%) | | 27 (67.50%) | | 44 (73.33%) | | 35 (72.92%) | | 12 (70.59%) | | |  | | |  | | |  |  |
| ***^2a^* After receiving my PGx results, I am more likely to follow the medication treatment recommended by my physician/pharmacist. (1 = Not at all, 5 = Absolutely)** | | | | | | | | | | | |  | | | 0.5 | | | >0.9 | | | |
| 2 | | 4 (1.99%) | 0 (0.00%) | | 2 (5.00%) | | 1 (1.67%) | | 0 (0.00%) | | 1 (5.88%) | | |  | | |  | | |  |  |
| 3 | | 15 (7.46%) | 2 (5.56%) | | 6 (15.00%) | | 2 (3.33%) | | 3 (6.25%) | | 2 (11.76%) | | |  | | |  | | |  |  |
| 4 | | 77 (38.31%) | 14 (38.89%) | | 13 (32.50%) | | 23 (38.33%) | | 20 (41.67%) | | 7 (41.18%) | | |  | | |  | | |  |  |
| 5 | | 105 (52.24%) | 20 (55.56%) | | 19 (47.50%) | | 34 (56.67%) | | 25 (52.08%) | | 7 (41.18%) | | |  | | |  | | |  |  |
| ***^2a^* I would recommend PGx testing to my children. (1 = Not at all, 5 = Absolutely)** | | | | | | | | | | | |  | | | >0.9 | | | >0.9 | | | |
| 3 | | 11 (5.47%) | 3 (8.33%) | | 3 (7.50%) | | 4 (6.67%) | | 1 (2.08%) | | 0 (0.00%) | | |  | | |  | | |  |  |
| 4 | | 86 (42.79%) | 14 (38.89%) | | 17 (42.50%) | | 25 (41.67%) | | 21 (43.75%) | | 9 (52.94%) | | |  | | |  | | |  |  |
| 5 | | 104 (51.74%) | 19 (52.78%) | | 20 (50.00%) | | 31 (51.67%) | | 26 (54.17%) | | 8 (47.06%) | | |  | | |  | | |  |  |
| ***^2a^* I would recommend PGx testing to a friend or relative. (1 = Not at all, 5 = Absolutely)** | | | | | | | | | | | |  | | | >0.9 | | | >0.9 | | | |
| 1 | | 1 (0.50%) | 0 (0.00%) | | 1 (2.50%) | | 0 (0.00%) | | 0 (0.00%) | | 0 (0.00%) | | |  | | |  | | |  |  |
| 3 | | 11 (5.47%) | 2 (5.56%) | | 3 (7.50%) | | 4 (6.67%) | | 2 (4.17%) | | 0 (0.00%) | | |  | | |  | | |  |  |
| 4 | | 87 (43.28%) | 15 (41.67%) | | 16 (40.00%) | | 24 (40.00%) | | 22 (45.83%) | | 10 (58.82%) | | |  | | |  | | |  |  |
| 5 | | 102 (50.75%) | 19 (52.78%) | | 20 (50.00%) | | 32 (53.33%) | | 24 (50.00%) | | 7 (41.18%) | | |  | | |  | | |  |  |
| ***^2a^* I am interested in participating in a future PGx study. (1 = Not at all, 5 = Absolutely)** | | | | | | | | | | | |  | | | 0.5 | | | >0.9 | | | |
| 1 | | 15 (7.46%) | 2 (5.56%) | | 2 (5.00%) | | 4 (6.67%) | | 4 (8.33%) | | 3 (17.65%) | | |  | | |  | | |  |  |
| 2 | | 18 (8.96%) | 3 (8.33%) | | 4 (10.00%) | | 8 (13.33%) | | 3 (6.25%) | | 0 (0.00%) | | |  | | |  | | |  |  |
| 3 | | 48 (23.88%) | 9 (25.00%) | | 8 (20.00%) | | 11 (18.33%) | | 15 (31.25%) | | 5 (29.41%) | | |  | | |  | | |  |  |
| 4 | | 60 (29.85%) | 6 (16.67%) | | 14 (35.00%) | | 19 (31.67%) | | 16 (33.33%) | | 5 (29.41%) | | |  | | |  | | |  |  |
| 5 | | 60 (29.85%) | 16 (44.44%) | | 12 (30.00%) | | 18 (30.00%) | | 10 (20.83%) | | 4 (23.53%) | | |  | | |  | | |  |  |
| ***^2a^* I made changes to my medication therapy on my own after receiving my PGx results, without physician guidance. (1 = Not at all, 5 = Absolutely)** | | | | | | | | | | | |  | | | >0.9 | | | >0.9 | | | |
| 1 | | 195 (97.01%) | 35 (97.22%) | | 40 (100.00%) | | 57 (95.00%) | | 46 (95.83%) | | 17 (100.00%) | | |  | | |  | | |  |  |
| 2 | | 1 (0.50%) | 0 (0.00%) | | 0 (0.00%) | | 1 (1.67%) | | 0 (0.00%) | | 0 (0.00%) | | |  | | |  | | |  |  |
| 3 | | 5 (2.49%) | 1 (2.78%) | | 0 (0.00%) | | 2 (3.33%) | | 2 (4.17%) | | 0 (0.00%) | | |  | | |  | | |  |  |
| ***^2a^* I made changes to my medication therapy after receiving my PGx results, based on my physician’s guidance. (1 = Not at all, 5 = Absolutely)** | | | | | | | | | | | |  | | | 0.3 | | | 0.9 | | | |
| 1 | | 105 (52.24%) | 20 (55.56%) | | 22 (55.00%) | | 32 (53.33%) | | 25 (52.08%) | | 6 (35.29%) | | |  | | |  | | |  |  |
| 2 | | 1 (0.50%) | 1 (2.78%) | | 0 (0.00%) | | 0 (0.00%) | | 0 (0.00%) | | 0 (0.00%) | | |  | | |  | | |  |  |
| 3 | | 18 (8.96%) | 3 (8.33%) | | 3 (7.50%) | | 2 (3.33%) | | 7 (14.58%) | | 3 (17.65%) | | |  | | |  | | |  |  |
| 4 | | 6 (2.99%) | 1 (2.78%) | | 3 (7.50%) | | 1 (1.67%) | | 0 (0.00%) | | 1 (5.88%) | | |  | | |  | | |  |  |
| 5 | | 71 (35.32%) | 11 (30.56%) | | 12 (30.00%) | | 25 (41.67%) | | 16 (33.33%) | | 7 (41.18%) | | |  | | |  | | |  |  |
| ***^2b^* I have noticed adverse drug reactions (ADRs) from a prescribed medication. (1 = Yes, 2 = No, 3 = I don’t remember)** | | | | | | | | | | | |  | | | 0.13 | | | 0.6 | | | |
| Yes | | 106 (52.74%) | 13 (36.11%) | | 24 (60.00%) | | 36 (60.00%) | | 26 (54.17%) | | 7 (41.18%) | | |  | | |  | | |  |  |
| No | | 95 (47.26%) | 23 (63.89%) | | 16 (40.00%) | | 24 (40.00%) | | 22 (45.83%) | | 10 (58.82%) | | |  | | |  | | |  |  |
| ***^2b^* Have you stopped taking a medication due to ineffectiveness? (1 = Yes, 2 = No, 3 = I don’t remember)** | | | | | | | | | | | |  | | | 0.030 | | | 0.2 | | | |
| Yes | | 103 (51.24%) | 12 (33.33%) | | 20 (50.00%) | | 40 (66.67%) | | 23 (47.92%) | | 8 (47.06%) | | |  | | |  | | |  |  |
| No | | 102 (48.76%) | 24 (66.67%) | | 20 (50.00%) | | 20 (33.33%) | | 25 (52.08%) | | 9 (52.94%) | | |  | | |  | | |  |  |
| ***^2a^* I am concerned about the confidentiality of my PGx testing results. (1 = Disagree, 4 = Agree)** | | | | | | | | | | | |  | | | 0.8 | | | >0.9 | | | |
| 1 | | 186 (92.54%) | 32 (88.89%) | | 37 (92.50%) | | 56 (93.33%) | | 44 (91.67%) | | 17 (100.00%) | | |  | | |  | | |  |  |
| 2 | | 14 (6.97%) | 3 (8.33%) | | 3 (7.50%) | | 4 (6.67%) | | 4 (8.33%) | | 0 (0.00%) | | |  | | |  | | |  |  |
| 3 | | 1 (0.50%) | 1 (2.78%) | | 0 (0.00%) | | 0 (0.00%) | | 0 (0.00%) | | 0 (0.00%) | | |  | | |  | | |  |  |
| ***^2a^* I am concerned that my PGx information could be used in future research without my permission. (1 = Disagree, 4 = Agree)** | | | | | | | | | | | |  | | | 0.4 | | | 0.9 | | | |
| 1 | | 186 (92.54%) | 32 (88.89%) | | 36 (90.00%) | | 57 (95.00%) | | 44 (91.67%) | | 17 (100.00%) | | |  | | |  | | |  |  |
| 2 | | 13 (6.47%) | 2 (5.56%) | | 4 (10.00%) | | 3 (5.00%) | | 4 (8.33%) | | 0 (0.00%) | | |  | | |  | | |  |  |
| 3 | | 2 (1.00%) | 2 (5.56%) | | 0 (0.00%) | | 0 (0.00%) | | 0 (0.00%) | | 0 (0.00%) | | |  | | |  | | |  |  |
| ***^2a^* I am generally satisfied with my experience using PGx testing. (0 = Not at all, 10 = Absolutely)** | | | | | | | | | | | |  | | | **0.024** | | | **0.2** | | | |
| 6 | | 3 (1.49%) | 2 (5.56%) | | 0 (0.00%) | | 1 (1.67%) | | 0 (0.00%) | | 0 (0.00%) | | |  | | |  | | |  |  |
| 7 | | 7 (3.48%) | 1 (2.78%) | | 4 (10.00%) | | 1 (1.67%) | | 1 (2.08%) | | 0 (0.00%) | | |  | | |  | | |  |  |
| 8 | | 34 (16.92%) | 10 (27.78%) | | 4 (10.00%) | | 14 (23.33%) | | 3 (6.25%) | | 3 (17.65%) | | |  | | |  | | |  |  |
| 9 | | 60 (29.85%) | 4 (11.11%) | | 12 (30.00%) | | 17 (28.33%) | | 21 (43.75%) | | 6 (35.29%) | | |  | | |  | | |  |  |
| 10 | | 97 (48.26%) | 19 (52.78%) | | 20 (50.00%) | | 27 (45.00%) | | 23 (47.92%) | | 8 (47.06%) | | |  | | |  | | |  |  |
|  |  |  |  |  |  |  |  |  |  |  |  |  |  |  | | |  |  |  |  |  |
| ***^2a^* PGx testing was helpful in making decisions about my health. (0 = Not at all, 10 = Absolutely)** | | | | | | | | | | | |  | | | 0.2 | | | 0.6 | | | |
| 5 | | 1 (0.50%) | 1 (2.78%) | | 0 (0.00%) | | 0 (0.00%) | | 0 (0.00%) | | 0 (0.00%) | | |  | | |  | | |  |  |
| 7 | | 16 (7.96%) | 4 (11.11%) | | 7 (17.50%) | | 3 (5.00%) | | 2 (4.17%) | | 0 (0.00%) | | |  | | |  | | |  |  |
| 8 | | 20 (9.95%) | 6 (16.67%) | | 6 (15.00%) | | 3 (5.00%) | | 3 (6.25%) | | 2 (11.76%) | | |  | | |  | | |  |  |
| 9 | | 52 (25.87%) | 8 (22.22%) | | 7 (17.50%) | | 19 (31.67%) | | 12 (25.00%) | | 6 (35.29%) | | |  | | |  | | |  |  |
| 10 | | 112 (55.72%) | 17 (47.22%) | | 20 (50.00%) | | 35 (58.33%) | | 31 (64.58%) | | 9 (52.94%) | | |  | | |  | | |  |  |
|  | *^1^* n (%) | | | | | | | | | | | | | | | | | | | |  |
|  | *^2a^* Fisher's Exact Test for Count Data with simulated p-value (based on 2000 replicates); *^2b^* Pearson's Chi-squared test | | | | | | | | | | | | | | | | | | | |  |
|  | *^3^* Benjamini & Hochberg correction for multiple testing | | | | | | | | | | | | | | | | | | | |  |

**Suppl. Table 6.** Impact of gender on participants’ answers. Note, two of the 28 questions in the questionnaire were not included in these comparisons due to zero variance in the provided responses. See Data Analysis section in the main manuscript. As a result, the total questions presented here are 26. The p-value and adjusted p-value refer to the comparison between the individual groups (Female, Male) and not between the groups and overall.

|  | | **Overall**, N = 201*^1^* | **Male**, N = 102*^1^* | **Female**, N = 99*^1^* | **p-value***^2^* | | **q-value***^3^* |  |
| --- | --- | --- | --- | --- | --- | --- | --- | --- |
| ***^2a^* I am familiar with PGx. (1 = Disagree, 4 = Agree)** | | | |  | 0.6 | | 0.8 |  |
| 1 | | 168 (83.58%) | 85 (83.33%) | 83 (83.84%) |  | |  |  |
| 2 | | 4 (1.99%) | 2 (1.96%) | 2 (2.02%) |  | |  |  |
| 3 | | 2 (1.00%) | 0 (0.00%) | 2 (2.02%) |  | |  |  |
| 4 | | 27 (13.43%) | 15 (14.71%) | 12 (12.12%) |  | |  |  |
| ***^2a^* DNA can affect my response to medications. (1 = Disagree, 4 = Agree)** | | | |  | 0.8 | | 0.8 |  |
| 1 | | 9 (4.48%) | 6 (5.88%) | 3 (3.03%) |  | |  |  |
| 2 | | 2 (1.00%) | 1 (0.98%) | 1 (1.01%) |  | |  |  |
| 3 | | 5 (2.49%) | 2 (1.96%) | 3 (3.03%) |  | |  |  |
| 4 | | 185 (92.04%) | 93 (91.18%) | 92 (92.93%) |  | |  |  |
| ***^2a^* I am aware of the existence of PGx. (1 = Disagree, 4 = Agree)** | | | |  | 0.8 | | 0.8 |  |
| 1 | | 149 (74.13%) | 77 (75.49%) | 72 (72.73%) |  | |  |  |
| 2 | | 20 (9.95%) | 9 (8.82%) | 11 (11.11%) |  | |  |  |
| 3 | | 10 (4.98%) | 4 (3.92%) | 6 (6.06%) |  | |  |  |
| 4 | | 22 (10.95%) | 12 (11.76%) | 10 (10.10%) |  | |  |  |
| ***^2a^* PGx testing can reduce the occurrence and severity of adverse drug reactions (ADRs). (1 = Disagree, 4 = Agree)** | | | |  | 0.8 | | 0.8 |  |
| 1 | | 1 (0.50%) | 1 (0.98%) | 0 (0.00%) |  | |  |  |
| 3 | | 3 (1.49%) | 1 (0.98%) | 2 (2.02%) |  | |  |  |
| 4 | | 197 (98.01%) | 100 (98.04%) | 97 (97.98%) |  | |  |  |
| ***^2b^* PGx testing can help identify the most adequate medication for me. (1 = Disagree, 4 = Agree)** | | | |  | 0.8 | | 0.8 |  |
| 3 | | 17 (8.46%) | 8 (7.84%) | 9 (9.09%) |  | |  |  |
| 4 | | 184 (91.54%) | 94 (92.16%) | 90 (90.91%) |  | |  |  |
| ***^2a^* PGx testing can reduce the frequency of relapse occurrence. (1 = Disagree, 4 = Agree)** | | | |  | 0.3 | | 0.8 |  |
| 2 | | 2 (1.00%) | 2 (1.96%) | 0 (0.00%) |  | |  |  |
| 3 | | 59 (29.35%) | 32 (31.37%) | 27 (27.27%) |  | |  |  |
| 4 | | 140 (69.65%) | 68 (66.67%) | 72 (72.73%) |  | |  |  |
| ***^2a^* PGx testing can contribute to the reduction of total healthcare expenditures. (1 = Disagree, 4 = Agree)** | | | |  | 0.6 | | 0.8 |  |
| 1 | | 1 (0.50%) | 0 (0.00%) | 1 (1.01%) |  | |  |  |
| 2 | | 23 (11.44%) | 11 (10.78%) | 12 (12.12%) |  | |  |  |
| 3 | | 82 (40.80%) | 39 (38.24%) | 43 (43.43%) |  | |  |  |
| 4 | | 95 (47.26%) | 52 (50.98%) | 43 (43.43%) |  | |  |  |
| ***^2a^* Would you prefer to have PGx testing before being prescribed the index drug? (1 = Disagree, 4 = Agree)** | | | |  | 0.2 | | 0.8 |  |
| 3 | | 2 (1.00%) | 0 (0.00%) | 2 (2.02%) |  | |  |  |
| 4 | | 199 (99.00%) | 102 (100.00%) | 97 (97.98%) |  | |  |  |
| ***^2a^* Would you like to learn more about PGx testing? (1 = Disagree, 4 = Agree)** | | | |  | 0.5 | | 0.8 |  |
| 1 | | 29 (14.43%) | 14 (13.73%) | 15 (15.15%) |  | |  |  |
| 2 | | 5 (2.49%) | 4 (3.92%) | 1 (1.01%) |  | |  |  |
| 3 | | 4 (1.99%) | 1 (0.98%) | 3 (3.03%) |  | |  |  |
| 4 | | 163 (81.09%) | 83 (81.37%) | 80 (80.81%) |  | |  |  |
| ***^2a^* Whose responsibility is to inform you and discuss your questions with based on your opinion?** | | | |  | 0.2 | | 0.8 |  |
| Physician | | 138 (68.66%) | 67 (65.69%) | 71 (71.72%) |  | |  |  |
| Researcher/Geneticist | | 59 (29.35%) | 31 (30.39%) | 28 (28.28%) |  | |  |  |
| Other | | 4 (1.99%) | 4 (3.92%) | 0 (0.00%) |  | |  |  |
| ***^2a^* When prescribed a new medication, it is important to inform my physician about my PGx results by showing my PGx card. (1 = Disagree, 4 = Agree)** | | | |  | 0.4 | | 0.8 |  |
| 1 | | 2 (1.00%) | 2 (1.96%) | 0 (0.00%) |  | |  |  |
| 2 | | 1 (0.50%) | 0 (0.00%) | 1 (1.01%) |  | |  |  |
| 4 | | 198 (98.51%) | 100 (98.04%) | 98 (98.99%) |  | |  |  |
| ***^2a^* I was properly informed about PGx before participating in this study. (1 = Disagree, 4 = Agree)** | | | |  | 0.5 | | 0.8 |  |
| 3 | | 1 (0.50%) | 0 (0.00%) | 1 (1.01%) |  | |  |  |
| 4 | | 200 (99.50%) | 102 (100.00%) | 98 (98.99%) |  | |  |  |
| ***^2b^* I believe that the benefits of PGx testing are clear. (1 = Disagree, 4 = Agree)** | | | |  | 0.5 | | 0.8 |  |
| 3 | | 43 (21.39%) | 24 (23.53%) | 19 (19.19%) |  | |  |  |
| 4 | | 158 (78.61%) | 78 (76.47%) | 80 (80.81%) |  | |  |  |
| ***^2b^* I trust more my physician who prescribes my medications.** | | | |  | 0.3 | | 0.8 |  |
| I don't know | | 32 (15.92%) | 20 (19.61%) | 12 (12.12%) |  | |  |  |
| No | | 25 (12.44%) | 13 (12.75%) | 12 (12.12%) |  | |  |  |
| Yes | | 144 (71.64%) | 69 (67.65%) | 75 (75.76%) |  | |  |  |
| ***^2a^* After receiving my PGx results, I am more likely to follow the medication treatment recommended by my physician/pharmacist. (1 = Not at all, 5 = Absolutely)** | | | |  | 0.7 | | 0.8 |  |
| 2 | | 4 (1.99%) | 1 (0.98%) | 3 (3.03%) |  | |  |  |
| 3 | | 15 (7.46%) | 9 (8.82%) | 6 (6.06%) |  | |  |  |
| 4 | | 77 (38.31%) | 40 (39.22%) | 37 (37.37%) |  | |  |  |
| 5 | | 105 (52.24%) | 52 (50.98%) | 53 (53.54%) |  | |  |  |
| ***^2b^* I would recommend PGx testing to my children. (1 = Not at all, 5 = Absolutely)** | | | |  | 0.15 | | 0.8 |  |
| 3 | | 11 (5.47%) | 7 (6.86%) | 4 (4.04%) |  | |  |  |
| 4 | | 86 (42.79%) | 49 (48.04%) | 37 (37.37%) |  | |  |  |
| 5 | | 104 (51.74%) | 46 (45.10%) | 58 (58.59%) |  | |  |  |
| ***^2a^* I would recommend PGx testing to a friend or relative. (1 = Not at all, 5 = Absolutely)** | | | |  | 0.10 | | 0.8 |  |
| 1 | | 1 (0.50%) | 1 (0.98%) | 0 (0.00%) |  | |  |  |
| 3 | | 11 (5.47%) | 7 (6.86%) | 4 (4.04%) |  | |  |  |
| 4 | | 87 (43.28%) | 50 (49.02%) | 37 (37.37%) |  | |  |  |
| 5 | | 102 (50.75%) | 44 (43.14%) | 58 (58.59%) |  | |  |  |
| ***^2b^* I am interested in participating in a future PGx study. (1 = Not at all, 5 = Absolutely)** | | | |  | 0.8 | | 0.8 |  |
| 1 | | 15 (7.46%) | 6 (5.88%) | 9 (9.09%) |  | |  |  |
| 2 | | 18 (8.96%) | 9 (8.82%) | 9 (9.09%) |  | |  |  |
| 3 | | 48 (23.88%) | 24 (23.53%) | 24 (24.24%) |  | |  |  |
| 4 | | 60 (29.85%) | 29 (28.43%) | 31 (31.31%) |  | |  |  |
| 5 | | 60 (29.85%) | 34 (33.33%) | 26 (26.26%) |  | |  |  |
| ***^2a^* I made changes to my medication therapy on my own after receiving my PGx results, without physician guidance. (1 = Not at all, 5 = Absolutely)** | | | |  | 0.4 | | 0.8 |  |
| 1 | | 195 (97.01%) | 98 (96.08%) | 97 (97.98%) |  | |  |  |
| 2 | | 1 (0.50%) | 0 (0.00%) | 1 (1.01%) |  | |  |  |
| 3 | | 5 (2.49%) | 4 (3.92%) | 1 (1.01%) |  | |  |  |
| ***^2a^* I made changes to my medication therapy after receiving my PGx results, based on my physician’s guidance. (1 = Not at all, 5 = Absolutely)** | | | |  | 0.3 | | 0.8 |  |
| 1 | | 105 (52.24%) | 60 (58.82%) | 45 (45.45%) |  | |  |  |
| 2 | | 1 (0.50%) | 0 (0.00%) | 1 (1.01%) |  | |  |  |
| 3 | | 18 (8.96%) | 8 (7.84%) | 10 (10.10%) |  | |  |  |
| 4 | | 6 (2.99%) | 3 (2.94%) | 3 (3.03%) |  | |  |  |
| 5 | | 71 (35.32%) | 31 (30.39%) | 40 (40.40%) |  | |  |  |
| ***^2b^* I have noticed adverse drug reactions (ADRs) from a prescribed medication. (1 = Yes, 2 = No, 3 = I don’t remember)** | | | |  | 0.4 | | 0.8 |  |
| Yes | | 106 (52.74%) | 51 (50.00%) | 55 (55.56%) |  | |  |  |
| No | | 95 (47.26%) | 51 (50.00%) | 44 (44.44%) |  | |  |  |
| **Have you stopped taking a medication due to ineffectiveness? (1 = Yes, 2 = No, 3 = I don’t remember)** | | | |  | 0.5 | | 0.8 |  |
| Yes | | 103 (51.24%) | 50 (49.02%) | 53 (53.54%) |  | |  |  |
| No | | 98 (48.76%) | 52 (51.98%) | 46 (46.46%) |  | |  |  |
| ***^2a^* I am concerned about the confidentiality of my PGx testing results. (1 = Disagree, 4 = Agree)** | | | |  | 0.3 | | 0.8 |  |
| 1 | | 186 (92.54%) | 92 (90.20%) | 94 (94.95%) |  | |  |  |
| 2 | | 14 (6.97%) | 9 (8.82%) | 5 (5.05%) |  | |  |  |
| 3 | | 1 (0.50%) | 1 (0.98%) | 0 (0.00%) |  | |  |  |
| ***^2a^* I am concerned that my PGx information could be used in future research without my permission. (1 = Disagree, 4 = Agree)** | | | |  | 0.6 | | 0.8 |  |
| 1 | | 186 (92.54%) | 93 (91.18%) | 93 (93.94%) |  | |  |  |
| 2 | | 13 (6.47%) | 7 (6.86%) | 6 (6.06%) |  | |  |  |
| 3 | | 2 (1.00%) | 2 (1.96%) | 0 (0.00%) |  | |  |  |
| ***^2a^* I am generally satisfied with my experience using PGx testing. (0 = Not at all, 10 = Absolutely)** | | | |  | 0.2 | | 0.8 |  |
| 6 | | 3 (1.49%) | 2 (1.96%) | 1 (1.01%) |  | |  |  |
| 7 | | 7 (3.48%) | 6 (5.88%) | 1 (1.01%) |  | |  |  |
| 8 | | 34 (16.92%) | 17 (16.67%) | 17 (17.17%) |  | |  |  |
| 9 | | 60 (29.85%) | 33 (32.35%) | 27 (27.27%) |  | |  |  |
| 10 | | 97 (48.26%) | 44 (43.14%) | 53 (53.54%) |  | |  |  |
| ***^2a^* PGx testing was helpful in making decisions about my health. (0 = Not at all, 10 = Absolutely)** | | | |  | 0.2 | | 0.8 |  |
| 5 | | 1 (0.50%) | 1 (0.98%) | 0 (0.00%) |  | |  |  |
| 7 | | 16 (7.96%) | 12 (11.76%) | 4 (4.04%) |  | |  |  |
| 8 | | 20 (9.95%) | 11 (10.78%) | 9 (9.09%) |  | |  |  |
| 9 | | 52 (25.87%) | 26 (25.49%) | 26 (26.26%) |  | |  |  |
| 10 | | 112 (55.72%) | 52 (50.98%) | 60 (60.61%) |  | |  |  |
|  | *^1^* n (%) | | | | |  | | |
|  | *^2a^* Fisher's exact test; *^2b^* Pearson's Chi-squared test | | | | |  |  |  |
|  | *^3^* Benjamini & Hochberg correction for multiple testing | | | | |  |  |  |

**Suppl. Table 7.** Impact of level of education on participants’ answers. Note, two of the 28 questions in the questionnaire were not included in these comparisons due to zero variance in the provided responses. See Data Analysis section in the main manuscript. As a result, the total questions presented here are 26. The p-value and adjusted p-value refer to the comparison between the individual educational background groups and not between the groups and overall.

|  | | **Overall**,  N = 201*^1^* | **Primary School**,  N = 52*^1^* | **Junior High School**,  N = 35*^1^* | **High School**,  N = 63*^1^* | **Bachelor or higher**,  N = 51*^1^* | **p-value***^2^* | | | | **q-value***^3^* | |  |
| --- | --- | --- | --- | --- | --- | --- | --- | --- | --- | --- | --- | --- | --- |
| ***^2a^* I am familiar with PGx. (1 = Disagree, 4 = Agree)** | | | | | |  | | 0.2 | | | | 0.8 | |
| 1 | | 168 (83.58%) | 44 (84.62%) | 32 (91.43%) | 51 (80.95%) | 41 (80.39%) |  | | | |  | |  |
| 2 | | 4 (1.99%) | 1 (1.92%) | 2 (5.71%) | 1 (1.59%) | 0 (0.00%) |  | | | |  | |  |
| 3 | | 2 (1.00%) | 1 (1.92%) | 0 (0.00%) | 0 (0.00%) | 1 (1.96%) |  | | | |  | |  |
| 4 | | 27 (13.43%) | 6 (11.54%) | 1 (2.86%) | 11 (17.46%) | 9 (17.65%) |  | | | |  | |  |
| ***^2a^* DNA can affect my response to medications. (1 = Disagree, 4 = Agree)** | | | | | |  | | 0.12 | | | | 0.8 | |
| 1 | | 9 (4.48%) | 4 (7.69%) | 0 (0.00%) | 3 (4.76%) | 2 (3.92%) |  | | | |  | |  |
| 2 | | 2 (1.00%) | 0 (0.00%) | 1 (2.86%) | 1 (1.59%) | 0 (0.00%) |  | | | |  | |  |
| 3 | | 5 (2.49%) | 4 (7.69%) | 0 (0.00%) | 0 (0.00%) | 1 (1.96%) |  | | | |  | |  |
| 4 | | 185 (92.04%) | 44 (84.62%) | 34 (97.14%) | 59 (93.65%) | 48 (94.12%) |  | | | |  | |  |
| ***^2a^* I am aware of the existence of PGx. (1 = Disagree, 4 = Agree)** | | | | | |  | | 0.3 | | | | 0.8 | |
| 1 | | 149 (74.13%) | 43 (82.69%) | 29 (82.86%) | 43 (68.25%) | 34 (66.67%) |  | | | |  | |  |
| 2 | | 20 (9.95%) | 3 (5.77%) | 4 (11.43%) | 5 (7.94%) | 8 (15.69%) |  | | | |  | |  |
| 3 | | 10 (4.98%) | 1 (1.92%) | 0 (0.00%) | 5 (7.94%) | 4 (7.84%) |  | | | |  | |  |
| 4 | | 22 (10.95%) | 5 (9.62%) | 2 (5.71%) | 10 (15.87%) | 5 (9.80%) |  | | | |  | |  |
| ***^2a^* PGx testing can reduce the occurrence and severity of adverse drug reactions (ADRs). (1 = Disagree, 4 = Agree)** | | | | | |  | | 0.3 | | | | 0.8 | |
| 1 | | 1 (0.50%) | 0 (0.00%) | 0 (0.00%) | 0 (0.00%) | 1 (1.96%) |  | | | |  | |  |
| 3 | | 3 (1.49%) | 1 (1.92%) | 0 (0.00%) | 0 (0.00%) | 2 (3.92%) |  | | | |  | |  |
| 4 | | 197 (98.01%) | 51 (98.08%) | 35 (100.00%) | 63 (100.00%) | 48 (94.12%) |  | | | |  | |  |
| ***^2a^* PGx testing can help identify the most adequate medication for me. (1 = Disagree, 4 = Agree)** | | | | | |  | | 0.6 | | | | 0.8 | |
| 3 | | 17 (8.46%) | 6 (11.54%) | 3 (8.57%) | 3 (4.76%) | 5 (9.80%) |  | | | |  | |  |
| 4 | | 184 (91.54%) | 46 (88.46%) | 32 (91.43%) | 60 (95.24%) | 46 (90.20%) |  | | | |  | |  |
| ***^2a^* PGx testing can reduce the frequency of relapse occurrence. (1 = Disagree, 4 = Agree)** | | | | | |  | | 0.080 | | | | 0.8 | |
| 2 | | 2 (1.00%) | 0 (0.00%) | 1 (2.86%) | 1 (1.59%) | 0 (0.00%) |  | | | |  | |  |
| 3 | | 59 (29.35%) | 20 (38.46%) | 11 (31.43%) | 11 (17.46%) | 17 (33.33%) |  | | | |  | |  |
| 4 | | 140 (69.65%) | 32 (61.54%) | 23 (65.71%) | 51 (80.95%) | 34 (66.67%) |  | | | |  | |  |
| ***^2a^* PGx testing can contribute to the reduction of total healthcare expenditures. (1 = Disagree, 4 = Agree)** | | | | | |  | | 0.5 | | | | 0.8 | |
| 1 | | 1 (0.50%) | 1 (1.92%) | 0 (0.00%) | 0 (0.00%) | 0 (0.00%) |  | | | |  | |  |
| 2 | | 23 (11.44%) | 9 (17.31%) | 4 (11.43%) | 3 (4.76%) | 7 (13.73%) |  | | | |  | |  |
| 3 | | 82 (40.80%) | 19 (36.54%) | 14 (40.00%) | 28 (44.44%) | 21 (41.18%) |  | | | |  | |  |
| 4 | | 95 (47.26%) | 23 (44.23%) | 17 (48.57%) | 32 (50.79%) | 23 (45.10%) |  | | | |  | |  |
| ***^2a^* Would you prefer to have PGx testing before being prescribed the index drug? (1 = Disagree, 4 = Agree)** | | | | | |  | | 0.7 | | | | 0.8 | |
| 3 | | 2 (1.00%) | 1 (1.92%) | 0 (0.00%) | 0 (0.00%) | 1 (1.96%) |  | | | |  | |  |
| 4 | | 199 (99.00%) | 51 (98.08%) | 35 (100.00%) | 63 (100.00%) | 50 (98.04%) |  | | | |  | |  |
| ***^2a^* Would you like to learn more about PGx testing? (1 = Disagree, 4 = Agree)** | | | | | |  | | 0.3 | | | | 0.8 | |
| 1 | | 29 (14.43%) | 8 (15.38%) | 6 (17.14%) | 5 (7.94%) | 10 (19.61%) |  | | | |  | |  |
| 2 | | 5 (2.49%) | 3 (5.77%) | 0 (0.00%) | 1 (1.59%) | 1 (1.96%) |  | | | |  | |  |
| 3 | | 4 (1.99%) | 2 (3.85%) | 1 (2.86%) | 0 (0.00%) | 1 (1.96%) |  | | | |  | |  |
| 4 | | 163 (81.09%) | 39 (75.00%) | 28 (80.00%) | 57 (90.48%) | 39 (76.47%) |  | | | |  | |  |
| ***^2a^* Whose responsibility is to inform you and discuss your questions with based on your opinion?** | | | | | |  | | **0.034** | | | | **0.8** | |
| Physician | | 138 (68.66%) | 43 (82.69%) | 24 (68.57%) | 38 (60.32%) | 33 (64.71%) |  | | | |  | |  |
| Researcher/Geneticist | | 59 (29.35%) | 7 (13.46%) | 11 (31.43%) | 23 (36.51%) | 18 (35.29%) |  | | | |  | |  |
| Other | | 4 (1.99%) | 2 (3.85%) | 0 (0.00%) | 2 (3.17%) | 0 (0.00%) |  | |  | | | |  |
| ***^2a^* When prescribed a new medication, it is important to inform my physician about my PGx results by showing my PGx card. (1 = Disagree, 4 = Agree)** | | | | | |  | | 0.3 | | | | 0.8 | |
| 1 | | 2 (1.00%) | 1 (1.92%) | 1 (2.86%) | 0 (0.00%) | 0 (0.00%) |  | | | |  | |  |
| 2 | | 1 (0.50%) | 0 (0.00%) | 0 (0.00%) | 0 (0.00%) | 1 (1.96%) |  | | | |  | |  |
| 4 | | 198 (98.51%) | 51 (98.08%) | 34 (97.14%) | 63 (100.00%) | 50 (98.04%) |  | | | |  | |  |
| ***^2a^* I was properly informed about PGx before participating in this study. (1 = Disagree, 4 = Agree)** | | | | | |  | | 0.4 | | | | 0.8 | |
| 3 | | 1 (0.50%) | 0 (0.00%) | 0 (0.00%) | 0 (0.00%) | 1 (1.96%) |  | | | |  | |  |
| 4 | | 200 (99.50%) | 52 (100.00%) | 35 (100.00%) | 63 (100.00%) | 50 (98.04%) |  | | | |  | |  |
| ***^2b^* I believe that the benefits of PGx testing are clear. (1 = Disagree, 4 = Agree)** | | | | | |  | | 0.6 | | | | 0.8 | |
| 3 | | 43 (21.39%) | 12 (23.08%) | 10 (28.57%) | 12 (19.05%) | 9 (17.65%) |  | | | |  | |  |
| 4 | | 158 (78.61%) | 40 (76.92%) | 25 (71.43%) | 51 (80.95%) | 42 (82.35%) |  | | | |  | |  |
| ***^2a^* I trust more my physician who prescribes my medications.** | | | | | |  | | 0.4 | | | | 0.8 | |
| I don't know | | 32 (15.92%) | 8 (15.38%) | 10 (28.57%) | 8 (12.70%) | 6 (11.76%) |  | | | |  | |  |
| No | | 25 (12.44%) | 6 (11.54%) | 2 (5.71%) | 9 (14.29%) | 8 (15.69%) |  | | | |  | |  |
| Yes | | 144 (71.64%) | 38 (73.08%) | 23 (65.71%) | 46 (73.02%) | 37 (72.55%) |  | | | |  | |  |
| ***^2a^* After receiving my PGx results, I am more likely to follow the medication treatment recommended by my physician/pharmacist. (1 = Not at all, 5 = Absolutely)** | | | | | |  | | 0.6 | | | | 0.8 | |
| 2 | | 4 (1.99%) | 0 (0.00%) | 1 (2.86%) | 1 (1.59%) | 2 (3.92%) |  | | | |  | |  |
| 3 | | 15 (7.46%) | 5 (9.62%) | 4 (11.43%) | 4 (6.35%) | 2 (3.92%) |  | | | |  | |  |
| 4 | | 77 (38.31%) | 23 (44.23%) | 12 (34.29%) | 26 (41.27%) | 16 (31.37%) |  | | | |  | |  |
| 5 | | 105 (52.24%) | 24 (46.15%) | 18 (51.43%) | 32 (50.79%) | 31 (60.78%) |  | | | |  | |  |
| ***^2a^* I would recommend PGx testing to my children. (1 = Not at all, 5 = Absolutely)** | | | | | |  | | 0.5 | | | | 0.8 | |
| 3 | | 11 (5.47%) | 2 (3.85%) | 4 (11.43%) | 2 (3.17%) | 3 (5.88%) |  | | | |  | |  |
| 4 | | 86 (42.79%) | 26 (50.00%) | 15 (42.86%) | 23 (36.51%) | 22 (43.14%) |  | | | |  | |  |
| 5 | | 104 (51.74%) | 24 (46.15%) | 16 (45.71%) | 38 (60.32%) | 26 (50.98%) |  | | | |  | |  |
| ***^2a^* I would recommend PGx testing to a friend or relative. (1 = Not at all, 5 = Absolutely)** | | | | | |  | | 0.3 | | | | 0.8 | |
| 1 | | 1 (0.50%) | 0 (0.00%) | 0 (0.00%) | 0 (0.00%) | 1 (1.96%) |  | | | |  | |  |
| 3 | | 11 (5.47%) | 2 (3.85%) | 4 (11.43%) | 2 (3.17%) | 3 (5.88%) |  | | | |  | |  |
| 4 | | 87 (43.28%) | 28 (53.85%) | 15 (42.86%) | 22 (34.92%) | 22 (43.14%) |  | | | |  | |  |
| 5 | | 102 (50.75%) | 22 (42.31%) | 16 (45.71%) | 39 (61.90%) | 25 (49.02%) |  | | | |  | |  |
| ***^2a^* I am interested in participating in a future PGx study. (1 = Not at all, 5 = Absolutely)** | | | | | |  | | 0.5 | | | | 0.8 | |
| 1 | | 15 (7.46%) | 3 (5.77%) | 3 (8.57%) | 2 (3.17%) | 7 (13.73%) |  | | | |  | |  |
| 2 | | 18 (8.96%) | 3 (5.77%) | 5 (14.29%) | 6 (9.52%) | 4 (7.84%) |  | | | |  | |  |
| 3 | | 48 (23.88%) | 17 (32.69%) | 7 (20.00%) | 13 (20.63%) | 11 (21.57%) |  | | | |  | |  |
| 4 | | 60 (29.85%) | 17 (32.69%) | 11 (31.43%) | 18 (28.57%) | 14 (27.45%) |  | | | |  | |  |
| 5 | | 60 (29.85%) | 12 (23.08%) | 9 (25.71%) | 24 (38.10%) | 15 (29.41%) |  | | | |  | |  |
| ***^2a^* I made changes to my medication therapy on my own after receiving my PGx results, without physician guidance. (1 = Not at all, 5 = Absolutely)** | | | | | |  | | 0.5 | | | | 0.8 | |
| 1 | | 195 (97.01%) | 52 (100.00%) | 34 (97.14%) | 59 (93.65%) | 50 (98.04%) |  | | | |  | |  |
| 2 | | 1 (0.50%) | 0 (0.00%) | 0 (0.00%) | 1 (1.59%) | 0 (0.00%) |  | | | |  | |  |
| 3 | | 5 (2.49%) | 0 (0.00%) | 1 (2.86%) | 3 (4.76%) | 1 (1.96%) |  | | | |  | |  |
| ***^2a^* I made changes to my medication therapy after receiving my PGx results, based on my physician’s guidance. (1 = Not at all, 5 = Absolutely)** | | | | | |  | | 0.7 | | | | 0.8 | |
| 1 | | 105 (52.24%) | 29 (55.77%) | 19 (54.29%) | 31 (49.21%) | 26 (50.98%) |  | | | |  | |  |
| 2 | | 1 (0.50%) | 0 (0.00%) | 0 (0.00%) | 0 (0.00%) | 1 (1.96%) |  | | | |  | |  |
| 3 | | 18 (8.96%) | 4 (7.69%) | 2 (5.71%) | 9 (14.29%) | 3 (5.88%) |  | | | |  | |  |
| 4 | | 6 (2.99%) | 2 (3.85%) | 1 (2.86%) | 3 (4.76%) | 0 (0.00%) |  | | | |  | |  |
| 5 | | 71 (35.32%) | 17 (32.69%) | 13 (37.14%) | 20 (31.75%) | 21 (41.18%) |  | | | |  | |  |
| ***^2a^* I have noticed adverse drug reactions (ADRs) from a prescribed medication. (1 = Yes, 2 = No, 3 = I don’t remember)** | | | | | |  | | 0.7 | | | | 0.8 | |
| Yes | | 106 (52.74%) | 26 (50.00%) | 21 (60.00%) | 34 (53.97%) | 25 (49.02%) |  | | | |  | |  |
| No | | 95 (47.26%) | 26 (50.00%) | 14 (40.00%) | 29 (46.03%) | 26 (50.98%) |  | | | |  | |  |
| ***^2b^* Have you stopped taking a medication due to ineffectiveness? (1 = Yes, 2 = No, 3 = I don’t remember)** | | | | | |  | | 0.7 | | | | 0.8 | |
| Yes | | 103 (51.24%) | 25 (48.08%) | 21 (60.00%) | 31 (49.21%) | 26 (50.98%) |  | | | |  | |  |
| No | | 98 (48.76%) | 27 (51.92%) | 14 (40.00%) | 32 (50.79%) | 25 (49.02%) |  | | | |  | |  |
| ***^2a^* I am concerned about the confidentiality of my PGx testing results. (1 = Disagree, 4 = Agree)** | | | | | |  | | 0.5 | | | | 0.8 | |
| 1 | | 186 (92.54%) | 50 (96.15%) | 33 (94.29%) | 59 (93.65%) | 44 (86.27%) |  | | | |  | |  |
| 2 | | 14 (6.97%) | 2 (3.85%) | 2 (5.71%) | 4 (6.35%) | 6 (11.76%) |  | | | |  | |  |
| 3 | | 1 (0.50%) | 0 (0.00%) | 0 (0.00%) | 0 (0.00%) | 1 (1.96%) |  | | | |  | |  |
| ***^2a^* I am concerned that my PGx information could be used in future research without my permission. (1 = Disagree, 4 = Agree)** | | | | | |  | | 0.7 | | | | 0.8 | |
| 1 | | 186 (92.54%) | 50 (96.15%) | 32 (91.43%) | 59 (93.65%) | 45 (88.24%) |  | | | |  | |  |
| 2 | | 13 (6.47%) | 2 (3.85%) | 3 (8.57%) | 3 (4.76%) | 5 (9.80%) |  | | | |  | |  |
| 3 | | 2 (1.00%) | 0 (0.00%) | 0 (0.00%) | 1 (1.59%) | 1 (1.96%) |  | | | |  | |  |
| ***^2a^* I am generally satisfied with my experience using PGx testing. (0 = Not at all, 10 = Absolutely)** | | | | | |  | | 0.8 | | | | 0.8 | |
| 6 | | 3 (1.49%) | 0 (0.00%) | 0 (0.00%) | 0 (0.00%) | 3 (5.88%) |  | | | |  | |  |
| 7 | | 7 (3.48%) | 1 (1.92%) | 1 (2.86%) | 3 (4.76%) | 2 (3.92%) |  | | | |  | |  |
| 8 | | 34 (16.92%) | 9 (17.31%) | 7 (20.00%) | 11 (17.46%) | 7 (13.73%) |  | | | |  | |  |
| 9 | | 60 (29.85%) | 18 (34.62%) | 12 (34.29%) | 18 (28.57%) | 12 (23.53%) |  | | | |  | |  |
| 10 | | 97 (48.26%) | 24 (46.15%) | 15 (42.86%) | 31 (49.21%) | 27 (52.94%) |  | | | |  | |  |
| ***^2a^* PGx testing was helpful in making decisions about my health. (0 = Not at all, 10 = Absolutely)** | | | | | |  | | 0.5 | | | | 0.8 | |
| 5 | | 1 (0.50%) | 0 (0.00%) | 0 (0.00%) | 0 (0.00%) | 1 (1.96%) |  | | | |  | |  |
| 7 | | 16 (7.96%) | 2 (3.85%) | 2 (5.71%) | 7 (11.11%) | 5 (9.80%) |  | | | |  | |  |
| 8 | | 20 (9.95%) | 5 (9.62%) | 4 (11.43%) | 6 (9.52%) | 5 (9.80%) |  | | | |  | |  |
| 9 | | 52 (25.87%) | 12 (23.08%) | 14 (40.00%) | 17 (26.98%) | 9 (17.65%) |  | | | |  | |  |
| 10 | | 112 (55.72%) | 33 (63.46%) | 15 (42.86%) | 33 (52.38%) | 31 (60.78%) |  | | | |  | |  |
|  | *^1^* n (%),*^2a^* Fisher's exact test; *^2b^* Pearson's Chi-squared test, *^3^* Benjamini & Hochberg correction for multiple testing | | | | | | | | |  | | | |

**Suppl. Table 8.** Impact of psychiatric indication on participants’ answers. Note, two of the 28 questions in the questionnaire were not included in these comparisons due to zero variance in the provided responses. See Data Analysis section in the main manuscript. As a result the total questions presented here are 26.

|  | | **Overall**, N = 201*^1^* | **Anxiety Disorders**, N = 7*^1^* | **Bipolar Disorder**, N = 40*^1^* | | **Major Depression**, N = 89*^1^* | | **Other**, N = 15*^1^* | | | **Schizophrenia**, N = 50*^1^* | | | **p-value***^2^* | | | **q-value***^3^* | |  |  |  |  |  |
| --- | --- | --- | --- | --- | --- | --- | --- | --- | --- | --- | --- | --- | --- | --- | --- | --- | --- | --- | --- | --- | --- | --- | --- |
| ***^2a^* I am familiar with PGx. (1 = Disagree, 4 = Agree)** | | | | | | | | |  | | | 0.2 | | | 0.4 | | | | |  |  |  |  |
| 1 | | 168 (83.58%) | 4 (57.14%) | 33 (82.50%) | | 73 (82.02%) | | 13 (86.67%) | | | 45 (90.00%) | | |  | | |  | |  |  |  |  |  |
| 2 | | 4 (1.99%) | 0 (0.00%) | 3 (7.50%) | | 1 (1.12%) | | 0 (0.00%) | | | 0 (0.00%) | | |  | | |  | |  |  |  |  |  |
| 3 | | 2 (1.00%) | 0 (0.00%) | 0 (0.00%) | | 1 (1.12%) | | 0 (0.00%) | | | 1 (2.00%) | | |  | | |  | |  |  |  |  |  |
| 4 | | 27 (13.43%) | 3 (42.86%) | 4 (10.00%) | | 14 (15.73%) | | 2 (13.33%) | | | 4 (8.00%) | | |  | | |  | |  |  |  |  |  |
| ***^2a^* DNA can affect my response to medications. (1 = Disagree, 4 = Agree)** | | | | | | | | |  | | | 0.3 | | | 0.5 | | | | |  |  |  |  |
| 1 | | 9 (4.48%) | 0 (0.00%) | 1 (2.50%) | | 5 (5.62%) | | 0 (0.00%) | | | 3 (6.00%) | | |  | | |  | |  |  |  |  |  |
| 2 | | 2 (1.00%) | 1 (14.29%) | 0 (0.00%) | | 0 (0.00%) | | 0 (0.00%) | | | 1 (2.00%) | | |  | | |  | |  |  |  |  |  |
| 3 | | 5 (2.49%) | 0 (0.00%) | 2 (5.00%) | | 1 (1.12%) | | 1 (6.67%) | | | 1 (2.00%) | | |  | | |  | |  |  |  |  |  |
| 4 | | 185 (92.04%) | 6 (85.71%) | 37 (92.50%) | | 83 (93.26%) | | 14 (93.33%) | | | 45 (90.00%) | | |  | | |  | |  |  |  |  |  |
| ***^2a^* I am aware of the existence of PGx. (1 = Disagree, 4 = Agree)** | | | | | | | | |  | | | 0.2 | | | 0.4 | | | | |  |  |  |  |
| 1 | | 149 (74.13%) | 4 (57.14%) | 32 (80.00%) | | 66 (74.16%) | | 13 (86.67%) | | | 34 (68.00%) | | |  | | |  | |  |  |  |  |  |
| 2 | | 20 (9.95%) | 0 (0.00%) | 4 (10.00%) | | 6 (6.74%) | | 0 (0.00%) | | | 10 (20.00%) | | |  | | |  | |  |  |  |  |  |
| 3 | | 10 (4.98%) | 0 (0.00%) | 1 (2.50%) | | 6 (6.74%) | | 1 (6.67%) | | | 2 (4.00%) | | |  | | |  | |  |  |  |  |  |
| 4 | | 22 (10.95%) | 3 (42.86%) | 3 (7.50%) | | 11 (12.36%) | | 1 (6.67%) | | | 4 (8.00%) | | |  | | |  | |  |  |  |  |  |
| ***^2a^* PGx testing can reduce the occurrence and severity of adverse drug reactions (ADRs). (1 = Disagree, 4 = Agree)** | | | | | | | | |  | | | 0.6 | | | 0.8 | | | | |  |  |  |  |
| 1 | | 1 (0.50%) | 0 (0.00%) | 0 (0.00%) | | 1 (1.12%) | | 0 (0.00%) | | | 0 (0.00%) | | |  | | |  | |  |  |  |  |  |
| 3 | | 3 (1.49%) | 0 (0.00%) | 2 (5.00%) | | 1 (1.12%) | | 0 (0.00%) | | | 0 (0.00%) | | |  | | |  | |  |  |  |  |  |
| 4 | | 197 (98.01%) | 7 (100.00%) | 38 (95.00%) | | 87 (97.75%) | | 15 (100.00%) | | | 50 (100.00%) | | |  | | |  | |  |  |  |  |  |
| ***^2a^* PGx testing can help identify the most adequate medication for me. (1 = Disagree, 4 = Agree)** | | | | | | | | |  | | | 0.8 | | | 0.9 | | | | |  |  |  |  |
| 3 | | 17 (8.46%) | 0 (0.00%) | 4 (10.00%) | | 6 (6.74%) | | 2 (13.33%) | | | 5 (10.00%) | | |  | | |  | |  |  |  |  |  |
| 4 | | 184 (91.54%) | 7 (100.00%) | 36 (90.00%) | | 83 (93.26%) | | 13 (86.67%) | | | 45 (90.00%) | | |  | | |  | |  |  |  |  |  |
| ***^2a^* PGx testing can reduce the frequency of relapse occurrence. (1 = Disagree, 4 = Agree)** | | | | | | | | |  | | | 0.058 | | | 0.3 | | | | |  |  |  |  |
| 2 | | 2 (1.00%) | 0 (0.00%) | 0 (0.00%) | | 0 (0.00%) | | 1 (6.67%) | | | 1 (2.00%) | | |  | | |  | |  |  |  |  |  |
| 3 | | 59 (29.35%) | 1 (14.29%) | 7 (17.50%) | | 26 (29.21%) | | 7 (46.67%) | | | 18 (36.00%) | | |  | | |  | |  |  |  |  |  |
| 4 | | 140 (69.65%) | 6 (85.71%) | 33 (82.50%) | | 63 (70.79%) | | 7 (46.67%) | | | 31 (62.00%) | | |  | | |  | |  |  |  |  |  |
| ***^2a^* PGx testing can contribute to the reduction of total healthcare expenditures. (1 = Disagree, 4 = Agree)** | | | | | | | | |  | | | **0.029** | | | **0.15** | | | | |  |  |  |  |
| 1 | | 1 (0.50%) | 0 (0.00%) | 1 (2.50%) | | 0 (0.00%) | | 0 (0.00%) | | | 0 (0.00%) | | |  | | |  | |  |  |  |  |  |
| 2 | | 23 (11.44%) | 0 (0.00%) | 2 (5.00%) | | 11 (12.36%) | | 4 (26.67%) | | | 6 (12.00%) | | |  | | |  | |  |  |  |  |  |
| 3 | | 82 (40.80%) | 4 (57.14%) | 12 (30.00%) | | 32 (35.96%) | | 5 (33.33%) | | | 29 (58.00%) | | |  | | |  | |  |  |  |  |  |
| 4 | | 95 (47.26%) | 3 (42.86%) | 25 (62.50%) | | 46 (51.69%) | | 6 (40.00%) | | | 15 (30.00%) | | |  | | |  | |  |  |  |  |  |
| ***^2a^* Would you prefer to have PGx testing before being prescribed the index drug? (1 = Disagree, 4 = Agree)** | | | | | | | | |  | | | 0.6 | | | 0.8 | | | | |  |  |  |  |
| 3 | | 2 (1.00%) | 0 (0.00%) | 1 (2.50%) | 1 (1.12%) | | 0 (0.00%) | | | 0 (0.00%) | | |  | | |  | |  |  |  |  |  |  |
| 4 | | 199 (99.00%) | 3 (100.00%) | 39 (97.50%) | 88 (98.88%) | | 19 (100.00%) | | | 50 (100.00%) | | |  | | |  | |  |  |  |  |  |  |
| ***^2a^* Would you like to learn more about PGx testing? (1 = Disagree, 4 = Agree)** | | | | | | | | |  | | | 0.8 | | | 0.9 | | | | |  |  |  |  |
| 1 | | 29 (14.43%) | 0 (0.00%) | 5 (12.50%) | | 13 (14.61%) | | 2 (13.33%) | | | 9 (18.00%) | | |  | | |  | |  |  |  |  |  |
| 2 | | 5 (2.49%) | 0 (0.00%) | 2 (5.00%) | | 1 (1.12%) | | 0 (0.00%) | | | 2 (4.00%) | | |  | | |  | |  |  |  |  |  |
| 3 | | 4 (1.99%) | 0 (0.00%) | 2 (5.00%) | | 2 (2.25%) | | 0 (0.00%) | | | 0 (0.00%) | | |  | | |  | |  |  |  |  |  |
| 4 | | 163 (81.09%) | 7 (100.00%) | 31 (77.50%) | | 73 (82.02%) | | 13 (86.67%) | | | 39 (78.00%) | | |  | | |  | |  |  |  |  |  |
| ***^2a^* Whose responsibility is to inform you and discuss your questions with based on your opinion?** | | | | | | | | |  | | | 0.8 | | | 0.9 | | | | |  |  |  |  |
| Physician | | 138 (68.66%) | 3 (42.86%) | 28 (70.00%) | | 62 (69.66%) | | 11 (73.33%) | | | 34 (68.00%) | | |  | | |  | |  |  |  |  |  |
| Researcher/Geneticist | | 59 (29.35%) | 4 (57.14%) | 11 (27.50%) | | 26 (29.21%) | | 4 (26.67%) | | | 14 (28.00%) | | |  | | |  | |  |  |  |  |  |
| Other | | 4 (1.99%) | 0 (0.00%) | 1 (2.50%) | | 1 (1.12%) | | 0 (0.00%) | | | 2 (4.00%) | | |  | | |  | |  |  |  |  |  |
| ***^2a^* When prescribed a new medication, it is important to inform my physician about my PGx results by showing my PGx card. (1 = Disagree, 4 = Agree)** | | | | | | | | |  | | | >0.9 | | | >0.9 | | | | |  |  |  |  |
| 1 | | 2 (1.00%) | 0 (0.00%) | 0 (0.00%) | | 1 (1.12%) | | 0 (0.00%) | | | 1 (2.00%) | | |  | | |  | |  |  |  |  |  |
| 2 | | 1 (0.50%) | 0 (0.00%) | 0 (0.00%) | | 1 (1.12%) | | 0 (0.00%) | | | 0 (0.00%) | | |  | | |  | |  |  |  |  |  |
| 4 | | 198 (98.51%) | 7 (100.00%) | 40 (100.00%) | | 87 (97.75%) | | 15 (100.00%) | | | 49 (98.00%) | | |  | | |  | |  |  |  |  |  |
| ***^2a^* I was properly informed about PGx before participating in this study. (1 = Disagree, 4 = Agree)** | | | | | | | | |  | | | >0.9 | | | >0.9 | | | | |  |  |  |  |
| 3 | | 1 (0.50%) | 0 (0.00%) | 0 (0.00%) | | 1 (1.12%) | | 0 (0.00%) | | | 0 (0.00%) | | |  | | |  | |  |  |  |  |  |
| 4 | | 200 (99.50%) | 7 (100.00%) | 40 (100.00%) | | 88 (98.88%) | | 15 (100.00%) | | | 50 (100.00%) | | |  | | |  | |  |  |  |  |  |
| ***^2a^* I believe that the benefits of PGx testing are clear. (1 = Disagree, 4 = Agree)** | | | | | | | | |  | | | 0.6 | | | 0.8 | | | | |  |  |  |  |
| 3 | | 43 (21.39%) | 1 (14.29%) | 6 (15.00%) | | 18 (20.22%) | | 4 (26.67%) | | | 14 (28.00%) | | |  | | |  | |  |  |  |  |  |
| 4 | | 158 (78.61%) | 6 (85.71%) | 34 (85.00%) | | 71 (79.78%) | | 11 (73.33%) | | | 36 (72.00%) | | |  | | |  | |  |  |  |  |  |
| ***^2a^* I trust more my physician who prescribes my medications.** | | | | | | | | |  | | | 0.13 | | | 0.4 | | | | |  |  |  |  |
| I don't know | | 32 (15.92%) | 0 (0.00%) | 6 (15.00%) | | 12 (13.48%) | | 6 (40.00%) | | | 8 (16.00%) | | |  | | |  | |  |  |  |  |  |
| No | | 25 (12.44%) | 0 (0.00%) | 4 (10.00%) | | 9 (10.11%) | | 1 (6.67%) | | | 11 (22.00%) | | |  | | |  | |  |  |  |  |  |
| Yes | | 144 (71.64%) | 7 (100.00%) | 30 (75.00%) | | 68 (76.40%) | | 8 (53.33%) | | | 31 (62.00%) | | |  | | |  | |  |  |  |  |  |
| ***^2a^* After receiving my PGx results, I am more likely to follow the medication treatment recommended by my physician/pharmacist. (1 = Not at all, 5 = Absolutely)** | | | | | | | | |  | | | **0.008** | | | **0.12** | | | | |  |  |  |  |
| 2 | | 4 (1.99%) | 0 (0.00%) | 0 (0.00%) | | 2 (2.25%) | | 0 (0.00%) | | | 2 (4.00%) | | |  | | |  | |  |  |  |  |  |
| 3 | | 15 (7.46%) | 1 (14.29%) | 1 (2.50%) | | 5 (5.62%) | | 0 (0.00%) | | | 8 (16.00%) | | |  | | |  | |  |  |  |  |  |
| 4 | | 77 (38.31%) | 0 (0.00%) | 15 (37.50%) | | 29 (32.58%) | | 8 (53.33%) | | | 25 (50.00%) | | |  | | |  | |  |  |  |  |  |
| 5 | | 105 (52.24%) | 6 (85.71%) | 24 (60.00%) | | 53 (59.55%) | | 7 (46.67%) | | | 15 (30.00%) | | |  | | |  | |  |  |  |  |  |
| ***^2a^* I would recommend PGx testing to my children. (1 = Not at all, 5 = Absolutely)** | | | | | | | | |  | | | **0.014** | | | **0.12** | | | | |  |  |  |  |
| 3 | | 11 (5.47%) | 0 (0.00%) | 0 (0.00%) | | 4 (4.49%) | | 0 (0.00%) | | | 7 (14.00%) | | |  | | |  | |  |  |  |  |  |
| 4 | | 86 (42.79%) | 3 (42.86%) | 15 (37.50%) | | 32 (35.96%) | | 9 (60.00%) | | | 27 (54.00%) | | |  | | |  | |  |  |  |  |  |
| 5 | | 104 (51.74%) | 4 (57.14%) | 25 (62.50%) | | 53 (59.55%) | | 6 (40.00%) | | | 16 (32.00%) | | |  | | |  | |  |  |  |  |  |
| ***^2a^* I would recommend PGx testing to a friend or relative. (1 = Not at all, 5 = Absolutely)** | | | | | | | | |  | | | **0.028** | | | **0.15** | | | | |  |  |  |  |
| 1 | | 1 (0.50%) | 0 (0.00%) | 0 (0.00%) | | 0 (0.00%) | | 0 (0.00%) | | | 1 (2.00%) | | |  | | |  | |  |  |  |  |  |
| 3 | | 11 (5.47%) | 0 (0.00%) | 0 (0.00%) | | 4 (4.49%) | | 1 (6.67%) | | | 6 (12.00%) | | |  | | |  | |  |  |  |  |  |
| 4 | | 87 (43.28%) | 3 (42.86%) | 15 (37.50%) | | 33 (37.08%) | | 9 (60.00%) | | | 27 (54.00%) | | |  | | |  | |  |  |  |  |  |
| 5 | | 102 (50.75%) | 4 (57.14%) | 25 (62.50%) | | 52 (58.43%) | | 5 (33.33%) | | | 16 (32.00%) | | |  | | |  | |  |  |  |  |  |
| ***^2a^* I am interested in participating in a future PGx study. (1 = Not at all, 5 = Absolutely)** | | | | | | | | |  | | | 0.5 | | | 0.7 | | | | |  |  |  |  |
| 1 | | 15 (7.46%) | 0 (0.00%) | 4 (10.00%) | | 5 (5.62%) | | 0 (0.00%) | | | 6 (12.00%) | | |  | | |  | |  |  |  |  |  |
| 2 | | 18 (8.96%) | 0 (0.00%) | 3 (7.50%) | | 4 (4.49%) | | 2 (13.33%) | | | 9 (18.00%) | | |  | | |  | |  |  |  |  |  |
| 3 | | 48 (23.88%) | 2 (28.57%) | 6 (15.00%) | | 24 (26.97%) | | 4 (26.67%) | | | 12 (24.00%) | | |  | | |  | |  |  |  |  |  |
| 4 | | 60 (29.85%) | 3 (42.86%) | 12 (30.00%) | | 28 (31.46%) | | 4 (26.67%) | | | 13 (26.00%) | | |  | | |  | |  |  |  |  |  |
| 5 | | 60 (29.85%) | 2 (28.57%) | 15 (37.50%) | | 28 (31.46%) | | 5 (33.33%) | | | 10 (20.00%) | | |  | | |  | |  |  |  |  |  |
| ***^2a^* I made changes to my medication therapy on my own after receiving my PGx results, without physician guidance. (1 = Not at all, 5 = Absolutely)** | | | | | | | | |  | | | 0.8 | | | 0.9 | | | | |  |  |  |  |
| 1 | | 195 (97.01%) | 7 (100.00%) | 38 (95.00%) | | 87 (97.75%) | | 15 (100.00%) | | | 48 (96.00%) | | |  | | |  | |  |  |  |  |  |
| 2 | | 1 (0.50%) | 0 (0.00%) | 0 (0.00%) | | 1 (1.12%) | | 0 (0.00%) | | | 0 (0.00%) | | |  | | |  | |  |  |  |  |  |
| 3 | | 5 (2.49%) | 0 (0.00%) | 2 (5.00%) | | 1 (1.12%) | | 0 (0.00%) | | | 2 (4.00%) | | |  | | |  | |  |  |  |  |  |
| ***^2a^* I made changes to my medication therapy after receiving my PGx results, based on my physician’s guidance. (1 = Not at all, 5 = Absolutely)** | | | | | | | | |  | | | 0.3 | | | 0.5 | | | | |  |  |  |  |
| 1 | | 105 (52.24%) | 5 (71.43%) | 20 (50.00%) | | 44 (49.44%) | | 10 (66.67%) | | | 26 (52.00%) | | |  | | |  | |  |  |  |  |  |
| 2 | | 1 (0.50%) | 0 (0.00%) | 0 (0.00%) | | 0 (0.00%) | | 0 (0.00%) | | | 1 (2.00%) | | |  | | |  | |  |  |  |  |  |
| 3 | | 18 (8.96%) | 0 (0.00%) | 1 (2.50%) | | 8 (8.99%) | | 2 (13.33%) | | | 7 (14.00%) | | |  | | |  | |  |  |  |  |  |
| 4 | | 6 (2.99%) | 0 (0.00%) | 4 (10.00%) | | 1 (1.12%) | | 0 (0.00%) | | | 1 (2.00%) | | |  | | |  | |  |  |  |  |  |
| 5 | | 71 (35.32%) | 2 (28.57%) | 15 (37.50%) | | 36 (40.45%) | | 3 (20.00%) | | | 15 (30.00%) | | |  | | |  | |  |  |  |  |  |
| ***^2b^* I have noticed adverse drug reactions (ADRs) from a prescribed medication. (1 = Yes, 2 = No, 3 = I don’t remember)** | | | | | | | | |  | | | 0.3 | | | 0.6 | | | | |  |  |  |  |
| Yes | | 106 (52.74%) | 5 (71.43%) | 24 (60.00%) | | 42 (47.19%) | | 6 (40.00%) | | | 29 (58.00%) | | |  | | |  | |  |  |  |  |  |
| No | | 95 (47.26%) | 2 (28.57%) | 16 (40.00%) | | 47 (52.81%) | | 9 (60.00%) | | | 21 (42.00%) | | |  | | |  | |  |  |  |  |  |
| ***^2b^* Have you stopped taking a medication due to ineffectiveness? (1 = Yes, 2 = No, 3 = I don’t remember)** | | | | | | | | |  | | | 0.2 | | | 0.4 | | | | |  |  |  |  |
| Yes | | 103 (51.24%) | 4 (57.14%) | 20 (50.00%) | | 42 (47.19%) | | 5 (33.33%) | | | 32 (64.00%) | | |  | | |  | |  |  |  |  |  |
| No | | 90 (48.76%) | 3 (42.86%) | 20 (50.00%) | | 47 (52.81%) | | 10 (66.667%) | | | 18 (36.00%) | | |  | | |  | |  |  |  |  |  |
| ***^2a^* I am concerned about the confidentiality of my PGx testing results. (1 = Disagree, 4 = Agree)** | | | | | | | | |  | | | 0.2 | | | 0.4 | | | | |  |  |  |  |
| 1 | | 186 (92.54%) | 7 (100.00%) | 35 (87.50%) | | 86 (96.63%) | | 15 (100.00%) | | | 43 (86.00%) | | |  | | |  | |  |  |  |  |  |
| 2 | | 14 (6.97%) | 0 (0.00%) | 5 (12.50%) | | 3 (3.37%) | | 0 (0.00%) | | | 6 (12.00%) | | |  | | |  | |  |  |  |  |  |
| 3 | | 1 (0.50%) | 0 (0.00%) | 0 (0.00%) | | 0 (0.00%) | | 0 (0.00%) | | | 1 (2.00%) | | |  | | |  | |  |  |  |  |  |
| ***^2a^* I am concerned that my PGx information could be used in future research without my permission. (1 = Disagree, 4 = Agree)** | | | | | | | | |  | | | 0.14 | | | 0.4 | | | | |  |  |  |  |
| 1 | | 186 (92.54%) | 6 (85.71%) | 36 (90.00%) | | 86 (96.63%) | | 15 (100.00%) | | | 43 (86.00%) | | |  | | |  | |  |  |  |  |  |
| 2 | | 2 (1.00%) | 1 (14.29%) | 4 (10.00%) | | 3 (3.37%) | | 0 (0.00%) | | | 5 (10.00%) | | |  | | |  | |  |  |  |  |  |
| 3 | | 13 (6.47%) | 0 (0.00%) | 0 (0.00%) | | 0 (0.00%) | | 0 (0.00%) | | | 2 (4.00%) | | |  | | |  | |  |  |  |  |  |
|  |  |  |  |  |  |  |  |  |  |  |  |  |  |  |  |  |  |  |  | | |  |  |
| **I am generally satisfied with my experience using PGx testing. (0 = Not at all, 10 = Absolutely)** | | | | | | | | |  | | | 0.2 | | | 0.4 | | | | |  | | |  |
| 6 | | 3 (1.49%) | 0 (0.00%) | 0 (0.00%) | | 1 (1.12%) | | 0 (0.00%) | | | 2 (4.00%) | | |  | | |  | |  | | |  |  |
| 7 | | 7 (3.48%) | 0 (0.00%) | 0 (0.00%) | | 2 (2.25%) | | 1 (6.67%) | | | 4 (8.00%) | | |  | | |  | |  | | |  |  |
| 8 | | 34 (16.92%) | 0 (0.00%) | 5 (12.50%) | | 12 (13.48%) | | 3 (20.00%) | | | 14 (28.00%) | | |  | | |  | |  | | |  |  |
| 9 | | 60 (29.85%) | 1 (14.29%) | 16 (40.00%) | | 26 (29.21%) | | 4 (26.67%) | | | 13 (26.00%) | | |  | | |  | |  | | |  |  |
| 10 | | 97 (48.26%) | 6 (85.71%) | 19 (47.50%) | | 48 (53.93%) | | 7 (46.67%) | | | 17 (34.00%) | | |  | | |  | |  | | |  |  |
| ***^2a^* PGx testing was helpful in making decisions about my health. (0 = Not at all, 10 = Absolutely)** | | | | | | | | |  | | | **0.013** | | | **0.12** | | | | |  | | |  |
| 5 | | 1 (0.50%) | 0 (0.00%) | 0 (0.00%) | | 1 (1.12%) | | 0 (0.00%) | | | 0 (0.00%) | | |  | | |  | |  | | |  |  |
| 7 | | 16 (7.96%) | 0 (0.00%) | 0 (0.00%) | | 6 (6.74%) | | 2 (13.33%) | | | 8 (16.00%) | | |  | | |  | |  | | |  |  |
| 8 | | 20 (9.95%) | 0 (0.00%) | 6 (15.00%) | | 3 (3.37%) | | 2 (13.33%) | | | 9 (18.00%) | | |  | | |  | |  | | |  |  |
| 9 | | 52 (25.87%) | 2 (28.57%) | 9 (22.50%) | | 22 (24.72%) | | 6 (40.00%) | | | 13 (26.00%) | | |  | | |  | |  | | |  |  |
| 10 | | 112 (55.72%) | 5 (71.43%) | 25 (62.50%) | | 57 (64.04%) | | 5 (33.33%) | | | 20 (40.00%) | | |  | | |  | |  | | |  |  |
|  |  |  |  |  |  |  |  |  |  |  |  |  |  |  |  |  |  |  |  | | |  |  |
|  | *^1^* n (%),*^2a^* Fisher's Exact Test for Count Data with simulated p-value (based on 2000 replicates); *^2b^* Pearson's Chi-squared test, *^3^* Benjamini & Hochberg correction for multiple testing | | | | | | | | | | | | | | | | | |  | |  | | |
|  |  | | | | | | | | | | | | | | | | | |  |  |  | | |

**Suppl. Figure 1.** Participants' responses towards informedness and behaviour. This group of questions includes one more question (I have all the necessary information to understand how PGx works as a pharmacotherapy tool), to which all patients have responded “Agree” and is thus not visualized here.

**
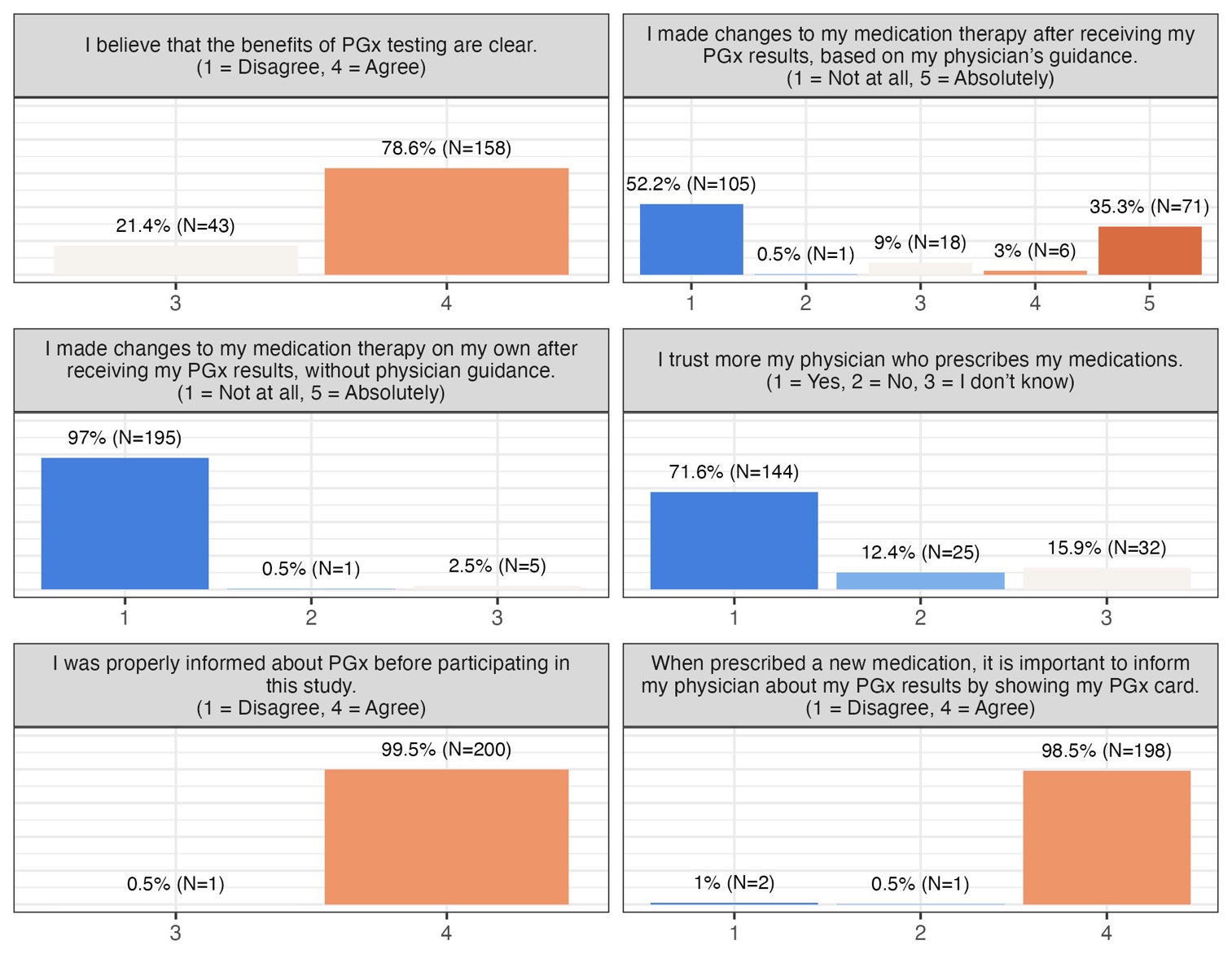
**
